# Disentangling Links Between Lung Cancer and Infectious Pneumonia via Real‐World Data and Integrative Genomics

**DOI:** 10.1155/humu/4536781

**Published:** 2026-01-31

**Authors:** Yi-Fei Diao, Zhe Chen, Yi-Fan Tang, Tian Xie, Qi-Yue Ge, Kai Xie, Zhuang-Zhuang Cong, Yi Shen

**Affiliations:** ^1^ Department of Cardiothoracic Surgery, Jinling Hospital, Affiliated Hospital of Medical School, Nanjing University, Nanjing, China, nju.edu.cn; ^2^ Department of Thoracic and Cardiovascular Surgery, The Fourth Affiliated Hospital of Soochow University, Suzhou Dushu Lake Hospital, Medical Center of Soochow University, Suzhou Key Laboratory of Chest Disease Diagnosis and Treatment, Suzhou, China; ^3^ Academy of Pharmacy, Xi′an Jiaotong–Liverpool University (XJTLU), Suzhou, China

**Keywords:** COPD, lung cancer, pneumonia, prognosis

## Abstract

Lung cancer (LC) patients frequently develop infectious pneumonia, often leading to suspension of anticancer therapy, yet the impact of LC on pneumonia progression remains unclear. This study employed a multidimensional approach to investigate whether LC constitutes a critical factor contributing to pulmonary infection onset and adverse short‐term outcomes. Data from two intensive care unit databases were analyzed to assess the association between LC and pneumonia incidence and prognosis from a real‐world perspective, with Mendelian randomization (MR) applied to validate causality. Additionally, post‐GWAS analyses were conducted to explore comorbidity interaction patterns and potential shared therapeutic targets. Cross‐sectional and cohort analyses identified LC as an independent risk factor for infectious pneumonia development and 28‐day mortality, findings corroborated by sensitivity analyses across multiple models and datasets. Meta‐analysis of MR results demonstrated causal relationships between genetically predicted LC and both pneumonia risk (OR = 1.103, 95% CI: 1.031–1.181, *p* = 0.004) and short‐term mortality (OR = 1.219, 95% CI: 1.100–1.350, *p* < 0.001), with consistency across histological subtypes. After adjustment for comorbidities including chronic obstructive pulmonary disease (COPD), LC retained independent effects, while a strong LC–COPD genetic correlation was observed. Subgroup and mediation analyses revealed a two‐way interplay between LC and COPD in driving pneumonia progression. Drug‐target analyses suggested that modulation of the complement and coagulation cascades may benefit pneumonia patients with comorbid LC or COPD, highlighting *CFB*, *SERPINA1*, and *SERPING1* as key candidates and pointing to monocyte‐centered pathways as promising therapeutic directions. These findings indicate that infection‐related pulmonary inflammation in LC patients may be partly tumor‐driven, challenging routine cessation of anticancer therapy and underscoring the need for comorbidity‐oriented treatment strategies.

## 1. Introduction

Lung cancer (LC) remains the leading cause of cancer‐related mortality worldwide [1]. Among its complications, infectious pneumonia poses a major threat to patient management and prognosis [2, 3]. From the clinical point of view, the occurrence of pneumonia in LC patients is by no means accidental, and its main mechanisms include tumor‐induced lung physiological dysfunction and abnormal immune regulation. Central‐type tumors or fast‐growing tumors can cause airway obstruction, affecting the mucus cilia clearance function, which in turn triggers obstructive pneumonia [4]. Meanwhile, immune dysfunction of the malignant tumor itself, coupled with the immunosuppressive effects of treatments such as chemotherapy, radiotherapy, targeted therapy, and immunotherapy, makes patients more susceptible to infectious pneumonia and immune‐related complications [5]. Although these mechanisms are recognized, most current studies focus on treatment‐related pneumonia, and evidence regarding infections directly attributable to LC is limited [6]. Moreover, observational studies are prone to confounding and reverse causation, making it difficult to establish a clear causal link between LC and pneumonia or its outcomes.

In clinical practice, physicians usually initiate anti‐infective therapy immediately while suspending antitumor therapy to avoid exacerbating the inflammatory response in the lungs [7]. However, this treatment faces a dilemma: Interruption of antitumor therapy may lead to tumor progression, while tumor progression itself may promote the development and worsening of lung infection. To further complicate matters, patients with LC combined with pneumonia often suffer from a variety of concurrent underlying conditions such as chronic obstructive pulmonary disease (COPD), diabetes, and heart failure [8, 9]. These comorbidities may individually or synergistically affect the patient′s prognosis, making clinical management even more problematic. This underscores the need for a deeper understanding of the interplay between LC, infectious pneumonia, and comorbidities to support evidence‐based clinical decision‐making.

To address these challenges, we conducted an integrative study combining real‐world data and genetic epidemiology. Using two large ICU databases, we assessed the association between LC and pneumonia through both cross‐sectional and longitudinal analyses. To overcome the inherent limitations of observational research, we applied Mendelian randomization (MR) to evaluate the causal effect of LC on pneumonia risk and 28‐day mortality [10]. By leveraging germline genetic variants as instrumental variables (IVs), MR offers a robust approach to reduce confounding and improve the validity of causal inference [11, 12]. To further account for the influence of comorbidities, we performed genetic correlation analysis and multivariable MR (MVMR) to explore shared genetic architecture and isolate the independent effect of LC. We also revealed important participatory features in the LC hazard effect through stratification and mediation analyses. Finally, we integrated protein quantitative trait loci (pQTL) and genome‐wide association study (GWAS) datasets to identify potential therapeutic targets and shared biological pathways across LC, pneumonia, and related comorbid conditions, and further assessed real‐world evidence, cell‐type–specific expression patterns, and druggable candidates that may inform comorbidity‐focused interventions.

Through triangulation of evidence from real‐world data and genetic analysis [13, 14], our findings consistently support a direct causal role of LC in the development of infectious pneumonia and its poor short‐term prognosis, and they also provide preliminary direction for future strategies aimed at targeting comorbidity pathways. This work highlights the complex relationship between cancer, infection, and comorbidity and calls for integrated treatment strategies balancing tumor control, infection management, and comorbidity care to improve outcomes in critically ill patients with LC.

## 2. Materials and Methods

### 2.1. Study Design

**Figure 1:**
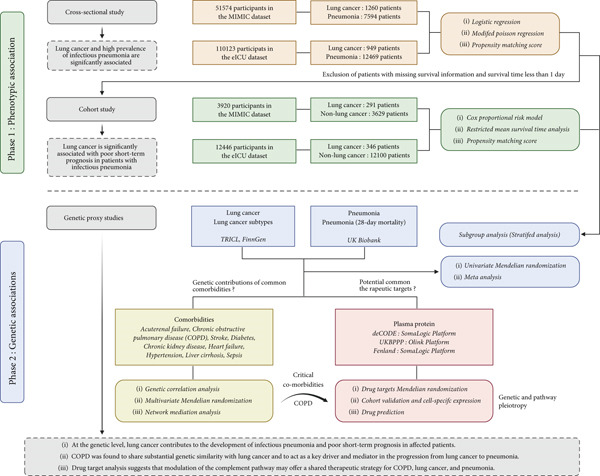
Research design and analysis flowchart. Overview of the study design integrating real‐world clinical data, genetic analyses, and drug‐target investigation. The first phase utilized the MIMIC and eICU datasets to assess the phenotypic associations between LC and infectious pneumonia and to evaluate their impact on 28‐day mortality through multiple statistical approaches and sensitivity analyses. The second phase used GWAS‐based MR to establish causality and characterize interaction and mediation effects driven by comorbidities. In addition, we incorporated drug‐target MR analysis, validation in external protein cohorts, single‐cell immune expression mapping, and drug enrichment analysis to further interpret the biological relevance of the identified targets. MIMIC: Medical Information Mart for Intensive Care; COPD: chronic obstructive pulmonary disease; GWAS: genome‐wide association study; MR: Mendelian randomization; LC: lung cancer.

This study integrated cross‐sectional, cohort, and post‐GWAS analyses to evaluate the impact of LC on the onset and short‐term outcomes of infectious pneumonia and to investigate the underlying genetic mechanisms. Figure 1 outlines the study design, and Supporting Information 1: Figure S1 details the post‐GWAS analytical workflow. All datasets used in this study were publicly available and accessed in compliance with their data use agreements. As the data were fully deidentified, neither informed consent nor institutional ethics approval was required. The design and analytical procedures of these datasets were rigorously developed in accordance with the STROBE (Strengthening the Reporting of Observational Studies in Epidemiology) reporting framework and the STROBE‐MR guidelines [15].

### 2.2. Retrospective Study

#### 2.2.1. Database and Study Population

Clinical data were obtained from two publicly available critical care databases: the Medical Information Mart for Intensive Care (MIMIC‐IV, Version 3.1) and the eICU Collaborative Research Database (eICU‐CRD). The MIMIC database contains detailed records of more than 90,000 critically ill patients admitted to Beth Israel Deaconess Medical Center between 2008 and 2022; the eICU database covers 208 US hospitals between 2014 and 2015 with 200,859 ICU hospitalizations. The investigator (Z.C.) received access to the MIMIC database after completing the Collaborating Institutions Training Program (Record No. 68706386). Patients were eligible for inclusion if they met all of the following criteria: (1) age ≥ 18 years, (2) first ICU admission, and (3) ICU stay ≥ 24 h.

#### 2.2.2. Patient Characteristics and Outcome Indicators

We extracted the following clinical baseline characteristics from the MIMIC database: (1) demographic factors: age, sex, and race and (2) disease‐related variables: comorbidities (hypertension, diabetes, stroke, COPD, heart failure, sepsis, liver cirrhosis, chronic kidney disease [CKD], and acute renal failure [ARF]) and clinical interventions (mechanical ventilation). Subsequently, we extracted the corresponding variables from the eICU database to maximize the reproduction of the analysis results.

In this study, LC characteristics were used as the primary exposure, and infectious pneumonia (hereafter referred to as pneumonia) and 28‐day mortality (including ICU mortality and in‐hospital mortality) were defined as the main outcomes. Detailed coding information is provided in Supporting Information 4: Table S1.

#### 2.2.3. Statistical Analysis

Continuous variables were compared using the Mann–Whitney *U* test, and categorical variables were analyzed using the chi‐square test. In cross‐sectional analyses, we assessed the association between LC and infectious pneumonia using logistic regression models to quantify the strength of the association in terms of the odds ratio (OR). Meanwhile, a modified Poisson regression model was used to calculate the prevalence ratio (PR) as an additional validation of the directionality and significance of the OR estimates. In the cohort analysis, we used the Cox proportional hazard model to estimate the relative risk of death between LC and non‐LC patients, expressed as hazard ratio (HR). Because the proportional hazard assumption may not fully hold for pneumonia‐related short‐term outcomes, we additionally applied restricted mean survival time (RMST) analysis as a complementary method. RMST quantifies the absolute difference in mean survival time as the area under the survival curve within a prespecified time window (RMST difference [RMSTd]), providing a robust and easily interpretable measure of survival that does not rely on the proportional hazard assumption [16]. By combining Cox and RMST approaches, we were able to capture both relative and absolute survival differences and ensure the robustness of our findings under potential nonproportional hazards. The above statistical tests were conducted using a two‐sided significance framework, and all analyses were completed in R software (Version 4.3.3). The MR and mediation analyses employed the following packages: TwoSampleMR (Version 0.6.15), MendelianRandomization (Version 0.7.0), MVMR (Version 0.3.2), boot (Version 1.3‐28) for bootstrap‐based mediation analysis, tidyverse (Version 2.0.0), data.table (Version 1.15.0) and dplyr (Version 1.1.4) for data cleaning and management, and coloc (Version 5.2.3) for colocalization analysis.

### 2.3. Post‐GWAS Analyses

#### 2.3.1. GWAS Data Sources

The GWAS datasets used in this study were obtained from three large research consortia: TRICL, FinnGen, and UK Biobank, with sample sizes ranging from 23,371 to 500,348 cases [17, 18]. TRICL and FinnGen also provided GWAS data for three histological subtypes of LC. GWAS data for comorbidities were derived from the FinnGen study, and data on infectious pneumonia and 28‐day pneumonia‐related mortality were obtained from the UK Biobank. For drug‐target analysis, we incorporated three large‐scale pQTL datasets from deCODE, UKB‐PPP, and Fenland [19–21]. Supporting Information 4: Table S2 provides detailed information on these datasets, including data code, sample sizes, ethnicity, and source studies.

#### 2.3.2. Genetic Correlation Analysis

We assessed genetic correlations between common comorbidities and multiple LC phenotypes using the linkage disequilibrium score regression (LDSC) method [22]. To verify the robustness of the results, we used high‐definition likelihood (HDL) analysis as a sensitivity assessment. HDL reduces the variance of genetic correlation estimates by approximately 60% compared to LDSC, resulting in improved precision and statistical validity [23].

#### 2.3.3. MR Analysis

The IVs were selected based on the following criteria: common variants with minor allele frequency > 0.01, genome‐wide significance (*p* < 5 × 10^−8^), and low linkage disequilibrium (*r* ^2^ < 0.001 within a 1000 kb window). For pneumonia‐related traits and FinnGen small cell lung cancer (SCLC), the significance threshold was relaxed to *p* < 5 × 10^−7^ due to limited power. SNPs showing strong associations with the outcome (*p* < 5 × 10^−7^) were excluded to ensure that valid causal inferences under independence were not violated. Instrument strength was evaluated using the *F*‐statistic calculated as *F* = *R* ^2^ × (*N* − 2)/(1 − *R* ^2^), with *R* ^2^ estimated from the expression [2 × EAF × (1 − EAF) × beta^2^]/{[2 × EAF × (1 − EAF) × beta^2^] + [2 × EAF × (1 − EAF) × *N* × SE^2^]}. In this equation, EAF denotes the effect allele frequency, beta is the SNP–exposure effect estimate, SE is its standard error, and *N* is the exposure GWAS sample size. An *F*‐statistic greater than 10 was considered evidence of sufficiently strong instruments [24].

Univariate MR (UVMR) heterogeneity was assessed using Cochran′s *Q* test when two or more IVs were available, while multivariable validity was evaluated using Egger′s intercept with three or more IVs. Directionality tests and reverse UVMR analyses were performed to confirm the correct causal direction. The Wald ratio method was applied when only one IV was available, and the inverse variance‐weighted (IVW) method with a random effects model was used for multiple instruments to account for heterogeneity. To further reduce potential bias from horizontal pleiotropy, the constrained maximum likelihood and model averaging (cML‐MA) method was employed [25]. This approach incorporates Bayesian information criterion (BIC) and data perturbation (DP) techniques, with model robustness evaluated through two goodness‐of‐fit tests. The cML‐MA‐BIC‐DP framework was applied when either test yielded *p* < 0.05. Given the possibility that smoking may introduce horizontal pleiotropy in the causal pathway between LC, pneumonia, and comorbidities, we additionally assessed whether any IVs overlapped with genetic signals of smoking behaviors. Using the genome‐wide significant SNPs (*p* < 5 × 10^−8^) from the smoking initiation GWAS by Liu et al., we identified and sequentially removed overlapping variants in a leave‐one‐out framework [26]. The stability of the MR estimates after exclusion of these SNPs was used to evaluate the extent to which smoking‐related pleiotropy could influence our findings. Causal estimates from MR analyses were expressed as ORs, representing the change in outcome risk per unit increase in exposure. For pQTL‐based exposures, ORs reflected the effect per standard deviation increase in genetically predicted protein levels.

MVMR was conducted using a sequential adjustment strategy to estimate the independent effects of multiple exposures on a single outcome, implemented with the “MVMR” package using exposure covariance matrices derived from LDSC [27]. To account for pleiotropy, the cML‐MA method implemented in the “MVMRcML” package was applied [28]. When Egger′s intercept indicated significant pleiotropy, results from the cML‐MA‐BIC‐DP approach were reported.

MR analyses were considered statistically significant at *p* < 0.05. In UVMR, associations passing Bonferroni correction (0.05 divided by the number of exposures multiplied by outcomes) were regarded as having strong causal evidence. MR estimates from different LC datasets were combined by meta‐analysis. Fixed effects results were reported as the main findings, with heterogeneity assessed across datasets. When *I* ^2^ > 50*%* and *p* < 0.05 and the fixed and random effects models yielded different results, estimates from the random effects model were also reported to ensure robustness.

#### 2.3.4. Mediation Analysis

Mediation effects were estimated using the coefficient product method based on two‐step UVMR [29], requiring statistically significant and directionally consistent effects in both steps (Supporting Information 2: Figure S2). Confidence intervals (CIs) and statistical significance were obtained through 10,000 bootstrap iterations [30], and the proportion of mediation was reported. Sensitivity analyses were further performed using the cML‐MA method to account for horizontal pleiotropy.

#### 2.3.5. Drug‐Target Analysis

Proteins, as key regulators of molecular pathways, are usually directly involved in the mechanisms of disease development and are therefore important targets for mechanistic studies and therapeutic interventions [31]. To investigate the causal roles of circulating proteins in disease outcomes, UVMR analyses were conducted using *cis*‐pQTLs (within ±100 kb of the encoding gene) as IVs to minimize horizontal pleiotropy, and pQTL‐GWAS pairs originating from the same national cohorts were excluded to reduce potential bias from sample overlap [32]. Proteins with significant causal effects were subjected to Kyoto Encyclopedia of Genes and Genomes (KEGG) pathway enrichment to identify relevant biological processes. Colocalization analyses were then performed based on the posterior probabilities of five hypotheses (PPH 0–4). A PPH4/(PPH3 + PPH4) ratio > 0.7 was considered a positive colocalization signal, PPH3 + PPH4 > 0.7 was considered supportive, and PPH4 > 0.7 indicated strong evidence of a shared causal variant within the *cis* region.

We further corroborated our findings by integrating external protein–trait association data from the UK Biobank and cell‐type–resolved expression profiles from the DICE (Database of Immune Cell Expression) single‐cell atlas [33, 34]. To explore potential therapeutic implications, drug‐target enrichment for the core proteins was performed using the Enrichr platform [35]. Together, these analyses offered independent validation and functional contextualization of the prioritized targets.

## 3. Results

### 3.1. Cross‐Sectional Study Correlation Analysis

A total of 51,574 hospitalized patients from the MIMIC database and 110,123 patients from the eICU database were included in this study. The median age of patients in the two groups was 67 years (interquartile range [IQR]: 55.00–78.00) and 65 years (IQR: 54.00–76.00), respectively, with a majority of male patients. Supporting Information 4: Table S3 and Supporting Information 4: Table S4 demonstrate in detail the comparison of demographic and clinical characteristics between groups.

In uncorrected analyses, both databases showed that the incidence of infectious pneumonia was significantly higher in LC patients than in non‐LC patients. The OR in the MIMIC database was 3.582 (95% CI: 3.133–4.095; *p* < 0.001), and a similar trend was observed in the eICU database (OR = 3.021, 95% CI: 2.665–3.424, *p* < 0.001), suggesting that this association was consistent and reproducible across populations. After correction for potential confounders, LC remained significantly associated with the occurrence of infectious pneumonia. The corrected OR was 3.947 (95% CI: 3.399–4.584, *p* < 0.001) for the MIMIC data and 2.366 (95% CI: 2.059–2.719, *p* < 0.001) for the eICU data. The modified Poisson regression model confirmed these findings, with all estimated PRs greater than 1 and statistically significant.

**Figure 2:**
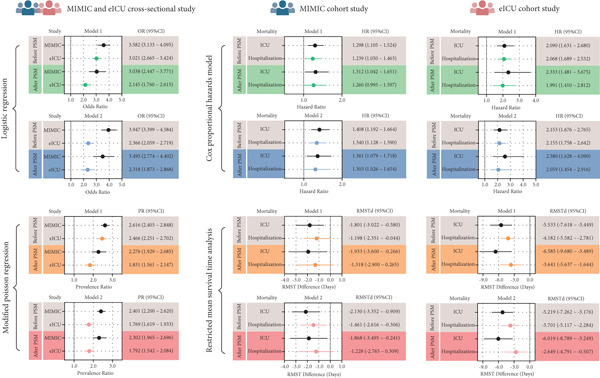
Forest map for a retrospective study of the effect of lung cancer on infectious pneumonia and its 28‐day mortality rate. MIMIC: Medical Information Mart for Intensive Care; PSM: propensity score matching; OR: odds ratio; PR: prevalence ratio; HR: hazard ratio; RMSTd: restricted mean survival time difference; CI: confidence interval.

To reduce potential bias from baseline differences, we performed PSM to achieve between‐group covariate balance. The results of the matching analysis were consistent with the main findings, further confirming the increased risk of infectious pneumonia in LC patients and validating the reliability of this association across different statistical methods and independent populations. The results of the sensitivity analyses are summarized and visualized in Figure 2 and Supporting Information 4: Table S5, Supporting Information 4: Table S6, Supporting Information 4: Table S7, Supporting Information 4: Table S8, Supporting Information 4: Table S9, and Supporting Information 4: Table S10.

### 3.2. Cohort Study Association

Given the elevated risk of in‐hospital death in patients with infectious pneumonia, we further explored whether LC affects short‐term prognosis in this population. We extracted data on all patients diagnosed with infectious pneumonia from both databases and excluded patients with missing survival information or survival time of less than 1 day to improve the accuracy and reliability of the survival analysis. A final total of 3920 patients from the MIMIC database and 12,446 patients from the eICU database were included. Baseline differences between the LC and non‐LC groups are shown in Supporting Information 4: Table S11 and Supporting Information 4: Table S12.

Before PSM in the MIMIC cohort, both univariate and multivariate Cox proportional risk models showed that LC was significantly associated with an increased risk of 28‐day ICU death (uncorrected HR = 1.298, 95% CI: 1.105–1.524, *p* = 0.001; corrected HR = 1.408, 95% CI: 1.192–1.664, *p* < 0.001). This association remained robust when the outcome was defined as in‐hospital death (uncorrected HR = 1.239, 95% CI: 1.050–1.463, *p* = 0.011; corrected HR = 1.340, 95% CI: 1.128–1.590, *p* < 0.001). Similar findings in the eICU cohort further confirmed LC as an independent risk factor for adverse short‐term outcomes in both ICU and hospital settings.

After PSM, baseline characteristics between groups were well balanced. Repeated Cox regression analyses yielded consistent results, supporting the association between LC and worse short‐term survival in patients with pneumonia. Although the RMST analysis in the matched MIMIC cohort did not reach statistical significance, all other analyses showed that LC was associated with reduced short‐term mean survival, suggesting that the coexistence of LC and pneumonia may lead to poorer clinical outcomes. Detailed results are presented in Figure 2 and Supporting Information 4: Table S13, Supporting Information 4: Table S14, Supporting Information 4: Table S15, Supporting Information 4: Table S16, Supporting Information 4: Table S17, and Supporting Information 4: Table S18.

### 3.3. Causal Association of LC With Infectious Pneumonia and Its Prognosis

To address the inherent limitations of observational analyses, we applied a genetic agent‐based UVMR framework to validate the associations. The SNP‐level *F*‐statistics for the instruments ranged from 25.521 to 96,878.048, with the vast majority exceeding the conventional threshold of 10. This indicates that the retained instruments were sufficiently strong to support reliable causal inference (Supporting Information 4: Table S19).

In the TRICL dataset, genetically predicted overall LC (OR = 1.122, 95% CI: 1.036–1.215, *p* = 0.005) and SCLC (OR = 1.122, 95% CI: 1.028–1.225, *p* = 0.010) were associated with an increased risk of pneumonia, while no significant associations were observed for non‐SCLC subtypes. In contrast, FinnGen data showed that lung adenocarcinoma (LUAD) (OR = 1.161, 95% CI: 1.073–1.256, *p* < 0.001) and lung squamous cell carcinoma (LUSC) (OR = 1.153, 95% CI: 1.077–1.235, *p* < 0.001) were significantly associated with increased pneumonia risk, while overall LC and SCLC did not show significant associations. Regarding short‐term prognosis, both datasets which demonstrated a significant association between overall LC (TRICL: OR = 1.204, 95% CI: 1.060–1.367, *p* = 0.004; FinnGen: OR = 1.248, 95% CI: 1.049–1.484, *p* = 0.013) and LUSC (TRICL: OR = 1.129, 95% CI: 1.012–1.260, *p* = 0.030; FinnGen: OR = 1.214, 95% CI: 1.016–1.450, *p* = 0.033) were associated with increased 28‐day mortality in patients with pneumonia. Additionally, FinnGen data showed a significant association between LUAD and short‐term mortality (OR = 1.291, 95% CI: 1.052–1.583, *p* = 0.014), but this finding was not validated in the TRICL dataset. Unlike the other subtypes, SCLC did not show a significant association with mortality in either data. Sensitivity analyses using the cML‐MA method produced consistent results in both effect direction and significance, aligning with estimates from the Wald ratio and IVW methods. The MR‐Egger intercept values ranged from −0.074 to 0.186, and all *p* values exceeded 0.05, suggesting limited bias from horizontal pleiotropy (Supporting Information 4: Table S20).

A meta‐analysis integrating the results of the two datasets further supported the presence of significant causal associations (*I* ^2^ range: 0.000%–79.086%), with pooled estimates largely concordant across fixed effect and random effects models. The pooled results indicated that genetic predictions of overall LC (OR = 1.103, 95% CI: 1.031–1.181, *p* = 0.004), LUAD (OR = 1.098, 95% CI: 1.044–1.155, *p* < 0.001), and LUSC (OR = 1.091, 95% CI: 1.041–1.144, *p* < 0.001) were all associated with an increased pneumonia risk. All associations exceeded the Bonferroni‐corrected significance threshold (*p* < 0.05/8). However, the association of LUSC with pneumonia did not reach statistical significance under the random effects model. SCLC showed a suggestive but nonsignificant association with the risk of pneumonia (OR = 1.096, 95% CI: 1.017–1.181, *p* = 0.016). Further pooled analysis showed that LC (OR = 1.219, 95% CI: 1.100–1.350, *p* < 0.001), LUSC (OR = 1.152, 95% CI: 1.049–1.264, *p* = 0.003), and LUAD (OR = 1.133, 95% CI: 1.028–1.249, *p* = 0.012) were significantly associated with an increased risk of pneumonia‐related short‐term mortality. However, after Bonferroni correction, only the association between overall LC and LUSC remained statistically significant, whereas the adverse prognostic effect of LUAD was not confirmed under the random effects model (Table 1 and Supporting Information 4: Table S21).

**Table 1:** The UVMR analysis results of LC characteristics on infectious pneumonia and its short‐term prognosis.

**Trait**	**TRICL**		**FinnGen**		**Meta**	
	**OR (95% CI)**	**p**	**OR (95% CI)**	**p**	**OR (95% CI)**	**p**
Pneumonia						
LC	1.122 (1.036~1.215)	0.005	1.057 (0.930~1.202)	0.395	1.103 (1.031~1.181)	0.004
LUAD	1.056 (0.988~1.128)	0.107	1.161 (1.073~1.256)	< 0.001	1.098 (1.044~1.155)	< 0.001
LUSC	1.037 (0.971~1.108)	0.274	1.153 (1.077~1.235)	< 0.001	1.091 (1.041~1.144)	< 0.001^a^
SCLC	1.122 (1.028~1.225)	0.010	1.030 (0.892~1.189)	0.687	1.096 (1.017~1.181)	0.016
Pneumonia 28‐day mortality						
LC	1.204 (1.060~1.367)	0.004	1.248 (1.049~1.484)	0.013	1.219 (1.100~1.350)	< 0.001
LUAD	1.091 (0.977~1.218)	0.121	1.291 (1.052~1.583)	0.014	1.133 (1.028~1.249)	0.012^a^
LUSC	1.129 (1.012~1.260)	0.030	1.214 (1.016~1.450)	0.033	1.152 (1.049~1.264)	0.003
SCLC	1.133 (0.835~1.537)	0.424	1.000 (0.784~1.276)	0.999	1.050 (0.868~1.270)	0.618

Abbreviations: CI, confidence interval; LC, lung cancer; LUAD, lung adenocarcinoma; LUSC, lung squamous cell carcinoma; OR, odds ratio; SCLC, small cell lung cancer; UVMR, univariable Mendelian randomization.

^a^Inconsistency with the fixed effects model was found in the random effects model.

Directionality tests supported a causal effect of LC on pneumonia outcomes, and reverse UVMR analysis found no evidence of reverse causality. Moreover, none of the instruments showed genome‐wide significant associations with smoking phenotype, and leave‐one‐out removal of valid SNPs did not materially alter the effect estimates (Supporting Information 7: Additional supporting information (available here)). These findings provide genetic support for a causal relationship between LC and both increased pneumonia risk and worse short‐term outcomes, while also highlighting subtype‐specific differences.

### 3.4. Genetic Correlation Analysis and Causal Associations Under Multiple Exposures

To investigate whether shared biological mechanisms between LC and common comorbidities might confound its effect on pneumonia, we conducted genetic correlation analyses using LDSC and HDL methods. To minimize sample overlap and ensure population independence, we integrated comorbidity data from FinnGen with LC data from TRICL and applied LDSC analysis with intercept correction. After Bonferroni correction (*p* < 0.05/40), significant genetic correlations were observed between COPD‐ and LC‐related traits, as well as between LUSC and sepsis (Figure 3a). Other comorbidities showed suggestive levels of genetic correlation with LC (Supporting Information 4: Table S22).

Figure 3Genetic associations of common comorbidities and LC and results of MVMR analysis. (a) Genetic correlation analysis of common comorbidities and LC. The double asterisk (∗∗) indicates significant associations after Bonferroni correction, and the single asterisk (∗) indicates suggestive associations. (b) Independent causal role of LC and its subtypes under MVMR analysis. The single asterisk (∗) indicates different findings between cML‐MA and IVW or Wald ratio methods. (c) Independent causal role of common comorbidities under MVMR analysis. The single asterisk (∗) indicates a *p* value < 0.05 for MVMR results. MVMR: multivariable Mendelian randomization; LC: lung cancer; LUAD: lung adenocarcinoma; LUSC: lung squamous cell carcinoma; SCLC: small cell lung cancer; ARF: acute renal failure; CKD: chronic kidney disease; T1D: Type 1 diabetes; T2D: Type 2 diabetes; COPD: chronic obstructive pulmonary disease; LDSC: linkage disequilibrium score regression; HDL: high‐definition likelihood; OR: odds ratio; CI: confidence interval; IVW: inverse variance‐weighted; cML‐MA: constrained maximum likelihood and model averaging.

**Figure:**
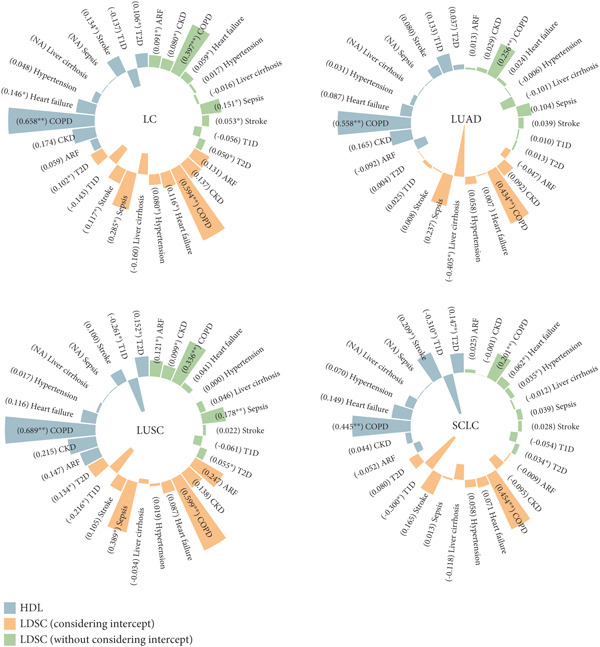
(a)

**Figure:**
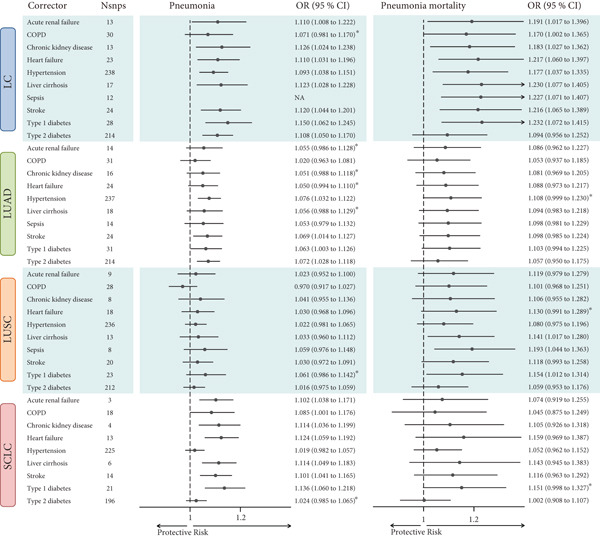
(b)

**Figure:**
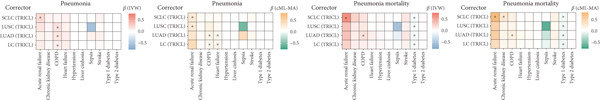
(c)

Given the potential for horizontal pleiotropy, MVMR was performed to assess the independent effect of LC on pneumonia risk and short‐term prognosis. Both univariate and multivariable analyses supported causal associations of ARF, CKD, COPD, and Type 1 diabetes with pneumonia. After adjusting for comorbidities, overall LC remained significantly associated with both pneumonia incidence and 28‐day mortality, except when accounting for sepsis in disease onset and Type 2 diabetes in prognosis (Supporting Information 4: Table S23 and Supporting Information 4: Table S24). Moreover, the associations between LC subtypes and the development and progression of pneumonia remained robust across multiple adjustment models.

Taken together, these results suggest that although there is a genetic overlap between LC and a variety of comorbidities, it has a limited impact on MR‐based causal inference. These results support the role of LC as an independent contributor to poor short‐term prognosis in patients with pneumonia (Figure 3b,c).

### 3.5. Integrated Analysis of Interaction and Mediation Effects

To clarify how common comorbidities participate in the effect of LC on pneumonia, we conducted an integrated analysis that focused on comorbidity‐related interaction patterns and potential mediation pathways. Cross‐sectional analyses of the MIMIC data revealed significant interactions between LC and heart failure, CKD, ARF, COPD, and sepsis in relation to pneumonia risk. In the eICU data, effect heterogeneity was primarily associated with COPD. The risk of pneumonia in patients with LC was lower among those with COPD than those without, and this heterogeneity remained evident in ICU and in‐hospital mortality. However, within the COPD subgroup, the association between LC and short‐term mortality was not statistically significant. A similar interaction was observed in patients with diabetes. Overall, COPD emerged as the most frequent source of effect heterogeneity, suggesting a key interaction pattern and potential biological similarity between COPD and LC and highlighting the importance of considering COPD status when evaluating outcomes in patients with coexisting LC and pneumonia (Figure 4a and Supporting Information 4: Table S25, Supporting Information 4: Table S26, Supporting Information 4: Table S27, Supporting Information 4: Table S28, Supporting Information 4: Table S29, and Supporting Information 4: Table S30).

Figure 4Subgroup analysis and mediation analysis. (a) Interaction plot of subgroup analysis results. (b) Mediation analysis result. OR: odds ratio; HR: hazard ratio; MIMIC: Medical Information Mart for Intensive Care; COPD: chronic obstructive pulmonary disease; LC: lung cancer; LUSC: lung squamous cell carcinoma.

**Figure:**
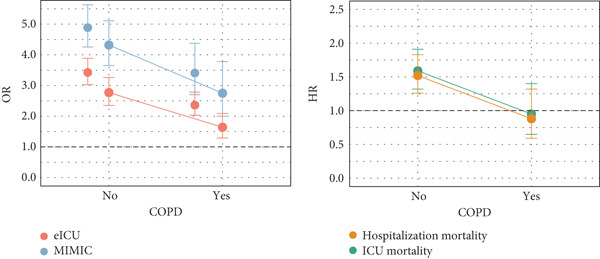
(a)

**Figure:**
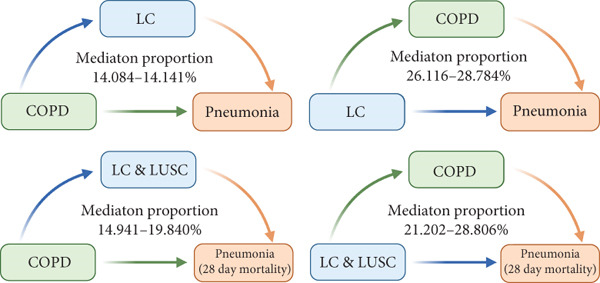
(b)

After identifying the main interaction factors, we further explored whether LC affected pneumonia through the common complication pathway. Bidirectional UVMR analyses revealed significant causal associations between LC and COPD, stroke, Type 1 diabetes, CKD, and hypertension, and these associations were validated using the cML‐MA method (Supporting Information 4: Table S31). After excluding associations with inconsistent directionality or failed directionality tests, a two‐step MR‐based mediation analysis was conducted. LC was found to mediate the effect of COPD on pneumonia risk, with mediation proportions ranging from 14.084% to 14.141%. Conversely, COPD also mediated the effect of LC on pulmonary inflammation, with a higher proportion (26.161%–28.784%). These bidirectional effects remained significant when 28‐day mortality was used as the outcome, with LUSC identified as a key subtype. In the leave‐one‐out analysis, two comorbidity‐related instruments (rs56222946 and rs2947411) were found to be associated with smoking behaviors. Removing these variants one at a time did not materially change the associations of COPD and Type 2 diabetes with the other phenotypes, supporting the robustness of our findings (Supporting Information 7: Additional supporting information). Overall, the findings support a reciprocal mediating relationship between LC and COPD in pneumonia pathogenesis, highlighting a shared comorbidity network among the three phenotypes (Figure 4b and Supporting Information 4: Table S32).

### 3.6. Common Targets in COPD, LC, and Pneumonia

Given the frequent co‐occurrence of COPD, LC, and pneumonia and their potential shared biological mechanisms, we investigated common causal drug targets at the genetic level. To avoid sample overlap bias, pQTL‐GWAS pairs from the same national source were excluded. Significant protein–trait associations were identified using the IVW or Wald ratio method and validated with the cML‐MA approach, yielding 5352 proteins with statistically significant causal effects (Figure 5a and Supporting Information 4: Table S33). Targets identified by both methods were subjected to pathway enrichment analysis, which revealed significant enrichment of the complement and coagulation cascade pathway across three disease traits (Figure 5b and Supporting Information 4: Table S34, Supporting Information 4: Table S35, and Supporting Information 4: Table S36), suggesting it may represent a core mechanism underlying the comorbidities.

Figure 5Drug‐target analysis in the comorbidity model. (a) Drug‐target analysis volcano diagram. (b) KEGG enrichment analysis of pneumonia features, lung cancer features, and COPD drug targets. (c) Summary plot of colocalization analyses. (d) Visualization of drug targets in pneumonia with strong colocalization evidence. (e) Heatmap of protein–trait association for the UK Biobank cohort. LC: lung cancer; LUAD: lung adenocarcinoma; LUSC: lung squamous cell carcinoma; SCLC: small cell lung cancer; COPD: chronic obstructive pulmonary disease; NSCLC: non–small cell lung cancer.

**Figure:**
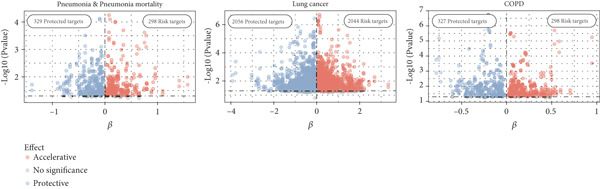
(a)

**Figure:**
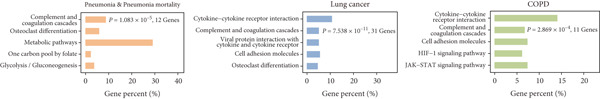
(b)

**Figure:**
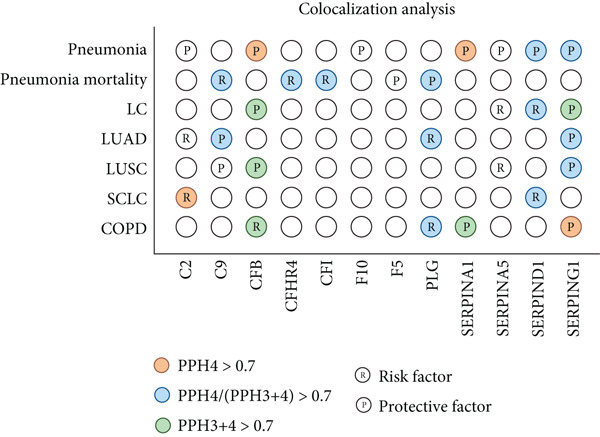
(c)

**Figure:**
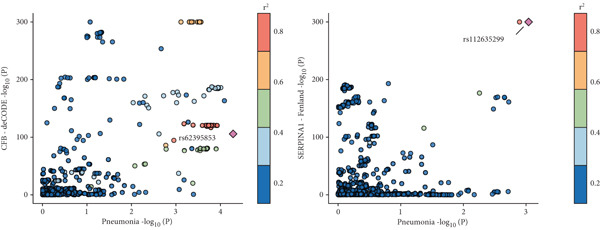
(d)

**Figure:**
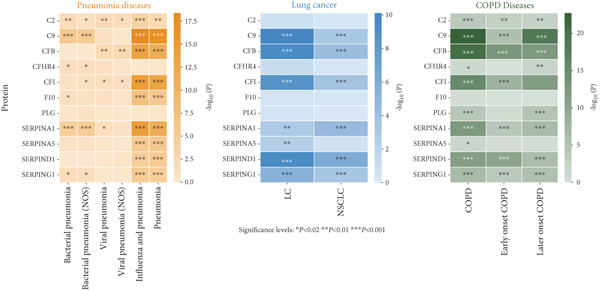
(e)

We then focused on proteins within this pathway that were significantly associated with pneumonia‐related traits and evaluated their pathogenic relevance in LC and COPD. Twelve proteins exerted genetic influences on pneumonia risk or 28‐day mortality (Figure 5c), and eight of these were also associated with LC or COPD after accounting for comorbidities. C2, C9, SERPINA5, and SERPIND1 were linked to LC risk. SERPINA1 was associated with COPD. CFB, PLG, and SERPING1 were associated with both conditions. To determine whether these associations arose from shared causal variants, we performed colocalization analyses (Supporting Information 4: Table S37). Eight pleiotropic protein targets received varying degrees of colocalization support, among which CFB (PPH4 [deCODE] = 0.751; PPH4 [Fenland] = 0.728) and SERPINA1 (PPH4 [Fenland] = 0.723) showed strong evidence of shared causal variants with pneumonia (Figure 5d). Additionally, C2 (PPH4 [Fenland] = 0.912) with SCLC (TRICL) and SERPING1 with COPD (PPH4 [deCODE] = 0.897) also demonstrated robust colocalization signals. These findings further support their roles as shared molecular drivers across the disease spectrum.

To further validate and extend the targets identified in our post‐GWAS analyses, we conducted independent protein–trait association analyses using UK Biobank data and assessed cell‐type–specific expression patterns using peripheral blood single‐cell RNA‐seq profiles. In the UK Biobank cohort, measurements were available for all complement proteins except F5, and each showed at least one significant association with pneumonia‐related, LC‐related, or COPD‐related phenotypes. With the exception of F10 and PLG, all proteins were linked to at least two phenotype categories, and notably, C9, CFB, CFI, SERPINA1, SERPINA5, SERPIND1, and SERPING1 were associated with phenotypes in all three disease domains (Figure 5e and Table S38). To delineate the cellular context of these associations, we analyzed single‐cell RNA‐seq data from immune cells. Differential expression analysis revealed that monocytes and B cells expressed 10 out of the 12 genes, followed by NK cells (9/12), and activated T cells (9/12). SERPINA1 was almost exclusively expressed in monocytes, with levels more than 1000‐fold higher than in other cell types (*p* < 0.001 for all comparisons). F5 was most highly expressed in activated T cells and regulatory T cells (> 20‐fold upregulation upon activation), with additional substantial expression in monocytes (all *p* < 0.001). Complement components (C2, C9, and CFB) and protease inhibitors (SERPING1 and SERPIND1) were predominantly expressed in monocytes (Supporting Information 3: Figure S3). Together, these findings indicate that monocytes are the principal cellular source of the complement and coagulation pathway proteins identified by our analyses and the cross‐phenotype associations highlight the central role of complement‐related targets in the comorbidity network linking LC, COPD, and pneumonia.

Drug enrichment analysis using DGIdb revealed that several existing therapeutics target the identified proteins. Notably, heparin was the most significantly enriched drug, interacting with both F10 and SERPINA5. Additional enriched agents targeting the coagulation–fibrinolysis axis included urokinase and lusutrombopag (PLG and F5), as well as hormonal modulators such as danazol and oxymetholone (Table S39). These findings suggest that repurposing existing anticoagulant and fibrinolytic agents may offer therapeutic opportunities for managing patients with pneumonia under comorbidities.

## 4. Discussion

This study systematically explored the relationship between LC and infectious pneumonia from multiple perspectives. Analyses based on two independent intensive care unit databases showed that LC was significantly associated with an increased risk of pneumonia and poor short‐term prognosis. MR analyses further supported these findings and suggested that different LC subtypes may have different causal effects. By integrating comorbidity‐associated genomic data, we identified genetic similarities between LC and a number of common diseases, particularly COPD. However, this genetic overlap did not substantially alter the independent causal effect of LC on pneumonia. Notably, we identified several potential causally mediation pathways, among which COPD appeared to be a key feature linking LC to the pneumonia phenotype. Further drug‐target analysis identified common pathogenic pathways and molecular targets that may be involved in COPD, LC, and pneumonia and revealed the cellular expression of these targets as well as drug enrichment. These findings deepen our understanding of disease interactions and provide new directions for synergistic therapeutic strategies.

The incidence of pneumonia in LC patients can be as high as 53% during antitumor therapy, and the mechanisms involved are often complex [36, 37]. Taking central LC as an example, it can cause airway obstruction and impair the mucus cilia clearance function, which makes patients prone to secondary infections and then develop serious complications such as pyothorax, lung abscess, or fistula formation [38]. In addition, tumor‐associated immunosuppression and malignancy significantly increase the susceptibility to opportunistic infections [39]. More importantly, a variety of antitumor treatments themselves can directly contribute to the development of pneumonia, including chemotherapy‐ or targeted therapy‐induced neutropenia, immune checkpoint inhibitor–associated immune‐mediated lung injury, treatment‐associated interstitial pneumonia, and radiation pneumonia [40–44]. Although previous studies have reported an increased incidence of pneumonia in patients with LC, high‐quality causal evidence remains lacking, and there is limited focus on short‐term prognosis in this population [45, 46]. In contrast, the present study used multiple analytical approaches to provide reliable evidence supporting the key causal role of LC in the development and progression of pneumonia. These findings highlight an important clinical dilemma. In current clinical practice, antitumor therapy is usually suspended during pneumonia episodes to reduce the risk of pulmonary toxicity [7]. However, such therapeutic interruptions may accelerate tumor progression and ultimately affect patient prognosis. This dual role exemplifies the inherent complexity of managing comorbidities. Our study provides strong evidence for this intricate relationship and provides a theoretical basis for the development of an integrated treatment strategy that balances tumor control with effective pneumonia management.

Genetic association analyses indicated that COPD may share more genetic loci with LC, which highlights the genetic basis for the combined treatment of COPD and LC. In addition, mediation analyses revealed a mutually reinforcing interaction between LC and COPD in promoting pneumonogenesis and increasing pneumonia‐related mortality. Previous MR studies exploring COPD and LC have usually failed to clearly distinguish the direction of causality, partly because of the difficulty in clarifying causality from clinical associations [47–49]. Although chronic lung inflammation in patients with COPD is known to increase the risk of lung malignancy over time [50], the limited survival time of LC patients may mask the development of subsequent COPD. Some investigators have suggested that LC may be associated with worsening COPD symptoms, but the causal relationship remains unclear [51]. Taken together, our findings suggest that COPD and LC may exert reinforcing bidirectional effects in promoting the development and progression of infectious pneumonia, providing a new theoretical basis for integrated therapeutic interventions.

Interestingly, in MR‐based survival analyses, after correction for LUAD, COPD was still significantly associated with a poor prognosis for pneumonia. However, when correcting for overall LC, the effect of COPD was no longer statistically significant, while LC itself remained associated with poor prognosis. This suggests that the effect of COPD may be moderated by specific LC subtypes. Although we were unable to determine that a particular subtype independently interacted with COPD to influence pneumonia prognosis, MVMR analysis suggested that LUSC may be most relevant, as its association was closer to statistical significance than other subtypes (OR = 1.101, 95*%*CI = 0.968–1.251, *p* = 0.142). In contrast, the results of the MVMR analysis of pneumonia risk suggested that SCLC may play a more independent role and be less affected by COPD. These findings suggest that interactions between COPD and different LC subtypes increase the complexity of lung inflammation and that the potentially independent effects of both diseases on pneumonia should not be overlooked. Given the frequent clinical coexistence of COPD and LC, the joint and independent contributions of these diseases must be carefully evaluated when managing pneumonia in patients with LC combined with COPD.

To enhance the translational relevance of this study, we found that complement and coagulation cascade pathways were consistently enriched in the short‐term adverse outcomes of LC, COPD, and infectious pneumonia, suggesting shared pathogenic mechanisms across these conditions. This finding is supported by our single‐cell transcriptomic analysis, which revealed that monocytes are the predominant cellular source of these pathway components, with SERPINA1 showing particularly striking monocyte‐specific expression. Such localization aligns with the established roles of monocyte‐derived C1q^+^ macrophages in tumor immunosurveillance, tumor progression, and pulmonary inflammatory responses [52, 53]. Complement involvement in infection‐related inflammation is further supported by evidence implicating C2 in childhood multisystem inflammatory syndrome and COVID‐19 [54, 55]. In our study, CFB and SERPINA1 showed genetically driven associations with pneumonia, indicating that shared germline variation may increase susceptibility to lung infections. This interpretation is consistent with experimental data showing that M28 family peptidases activate trained immunity against methicillin‐resistant *Staphylococcus aureus* through the CFB–C3a–C3aR–HIF1*α* pathway [56] and with observations in CFB‐deficient mouse models demonstrating enhanced bacterial dissemination and worsened inflammatory responses [57]. CFB has also been reported to suppress LC progression by downregulating the Ras/MAPK pathway, consistent with our protein–disease directionality [58]. The identification of existing anticoagulant agents, particularly heparin, as potential therapeutic candidates is noteworthy, as heparin exhibits anti‐inflammatory and immunomodulatory effects beyond its anticoagulant properties, including inhibition of proinflammatory cytokine expression in macrophages and NF‐*κ*B pathway suppression in alveolar epithelial cells [59]. These findings resonate with emerging evidence that thromboinflammation, characterized by the interplay between coagulation activation and inflammatory responses, represents a common pathophysiological mechanism in pulmonary diseases including severe pneumonia and cancer‐associated coagulopathy [60, 61]. Together, our single‐cell and drug enrichment analyses provide additional layers of biological plausibility for the identified comorbidity network and suggest that therapeutic strategies targeting complement–coagulation crosstalk may offer synergistic benefits in managing pneumonia among patients with LC and COPD comorbidities.

This study has several methodological advantages that enhance the reliability of the findings. First, comprehensive high‐resolution clinical data from the MIMIC and eICU databases enabled retrospective analyses that were highly comparable to real‐world clinical practice. Second, the MR method effectively reduces the effects of confounders and reverse causality, which are common in observational studies, thus enhancing the reliability of causal inferences. In addition, genetic correlation analysis, mediator model construction, and drug‐target localization together revealed complex mechanistic interactions in the comorbidity network, further enhancing the interpretability and translational application value of the study findings.

However, there are some limitations of this study. Although MR is a powerful tool for studying causality, it is still susceptible to horizontal pleiotropy and may introduce bias even after rigorous sensitivity testing. Furthermore, although we used PSM and multiple sensitivity analyses to minimize these effects, retrospective designs can still lead to information bias and selection bias. Further, our genetic analyses were primarily based on populations of European ancestry, limiting the generalizability of the findings. Furthermore, given the complex pattern of comorbidities involved in this study, the actual therapeutic effects of the identified core targets in comorbidity management have not been fully established. Future studies should incorporate experimental interventions and explore strategies that consider potential COPD interactions while balancing infection control with ongoing antitumor therapy to guide clinical decision‐making and improve the prognosis of patients with LC comorbidities.

## 5. Conclusion

In summary, this study provides novel and robust evidence that LC is causally associated with an increased risk of infectious pneumonia and poorer short‐term survival, based on real‐world and genetic data. It also reveals the critical interactive role of COPD in this process and identifies potential shared therapeutic targets across LC, COPD, and pneumonia. These findings highlight the importance of addressing key comorbidities in patient management and support the clinical value of integrating anti‐infective and anticancer treatments. Implementing such strategies may enable more precise and comprehensive care for patients with complex comorbid conditions.

**Nomenclature**
LC: lung cancer
MIMIC: Medical Information Mart for Intensive Care
MR: Mendelian randomization
post‐GWAS: post–genome‐wide association study
COPD: chronic obstructive pulmonary disease
LUSC: lung squamous cell carcinoma
IVs: instrumental variables
PSM: propensity score matching
SMD: standardized mean difference
RMST: restricted mean survival time
HR: hazard ratio
RMSTd: RMST difference
LDSC: linkage disequilibrium score regression
HDL: high‐definition likelihood
MAF: minor allele frequency
UVMR: univariable Mendelian randomization
IVW: inverse variance‐weighted
cML‐MA: constrained maximum likelihood and model averaging
BIC: Bayesian information criterion
DP: data perturbation
GOF: goodness‐of‐fit
OR: odds ratio
MVMR: multivariable Mendelian randomization
IQR: interquartile range

## Ethics Statement

This study was conducted using publicly available, deidentified datasets, including the Medical Information Mart for Intensive Care (MIMIC) database and genome‐wide association study (GWAS) summary statistics. Access to the MIMIC database was obtained following completion of the required data use agreement and associated training, which ensures strict adherence to ethical guidelines, including the prohibition of any attempt at reidentification. The GWAS datasets employed in this study were fully anonymized and accessible in the public domain and therefore did not require additional institutional review board (IRB) approval. All data analyses were performed in accordance with the relevant ethical standards and data usage policies.

## Conflicts of Interest

The authors declare no conflicts of interest.

## Author Contributions

The study was conceived and designed by Y‐F.D., Z.C., K.X., Z‐Z.C., and Y.S. Data curation and analysis were performed by Y‐F.D., Y‐F.T., K.X., Y.S., and Z‐Z.C. The analysis was supervised by Y‐F.D., K.X., and Y.S. Data interpretation was carried out by Z.C., T.X., Q‐Y.G., and Y‐F.D. The original draft was written and subsequently edited by all authors, who also approved the final version. Y‐F.D., Z.C., and Y‐F.T. contributed equally to this work and should be considered co‐first authors.

## Funding

This work was funded by the National Natural Science Foundation of China (82002454) and the Medical Scientific Research Project of Jiangsu Health Commission (ZD2021011).

## Supporting Information

Additional supporting information can be found online in the Supporting Information section.

## Supporting information


**Supporting Information 1** Figure S1: Mendelian randomization principles and mediation analysis process.

**Supporting Information 2** Figure S2: Post‐GWAS analysis process.

**Supporting Information 3** Figure S3: Violin diagram of expression pattern and differential analysis of potential comorbidity targets in peripheral blood immune cells.

**Supporting Information 4** Table S1: Code information for lung cancer and infectious pneumonia. Table S2: The GWAS data information utilized in the study. Table S3: Baseline characteristics of patients before PSM in the cross‐sectional study (MIMIC). Table S4: Baseline characteristics of patients before PSM in the cross‐sectional study (eICU). Table S5: Baseline characteristics of patients after PSM in the cross‐sectional study (MIMIC). Table S6: Baseline characteristics of patients after PSM in the cross‐sectional study (eICU). Table S7: Logistic regression results in the cross‐sectional study (MIMIC). Table S8: Logistic regression results in the cross‐sectional study (eICU). Table S9: Modified Poisson regression results in the cross‐sectional study (MIMIC). Table S10: Modified Poisson regression results in the cross‐sectional study (eICU). Table S11: Baseline characteristics of patients before PSM in the cohort study (MIMIC). Table S12: Baseline characteristics of patients before PSM in the cohort study (eICU). Table S13: Baseline characteristics of patients after PSM in the cohort study (MIMIC). Table S14: Baseline characteristics of patients after PSM in the cohort study (eICU). Table S15: Cox proportional hazard model results in the cohort study (MIMIC). Table S16: Cox proportional hazard model results in the cohort study (eICU). Table S17: RMST results in the cohort study (MIMIC). Table S18: RMST results in the cohort study (eICU). Table S19: Information on instrumental variables used in MR analysis. Table S20: UVMR analysis results of lung cancer and pneumonia. Table S21: Metaresults of UVMR analysis results of lung cancer and pneumonia. Table S22: Genetic correlation analysis of disease‐related variables and lung cancer. Table S23: UVMR analysis results of disease‐related variables and pneumonia. Table S24: MVMR analysis results. Table S25: Logistic regression subgroup analysis in the cross‐sectional study (MIMIC). Table S26: Logistic regression subgroup analysis in the cross‐sectional study (eICU). Table S27: Cox proportional hazard model subgroup analysis in the cohort study (MIMIC—ICU mortality). Table S28: Cox proportional hazard model subgroup analysis in the cohort study (MIMIC—hospitalization mortality). Table S29: Cox proportional hazard model subgroup analysis in the cohort study (eICU—ICU mortality). Table S30: Cox proportional hazard model subgroup analysis in the cohort study (eICU—hospitalization mortality). Table S31: UVMR analysis results of disease‐related variables and lung cancer. Table S32: Mediation MR analysis results. Table S33: Drug‐target MR analysis results. Table S34: Drug‐target enrichment analysis for pneumonia. Table S35: Drug‐target enrichment analysis for lung cancer. Table S36: Drug‐target enrichment analysis for COPD. Table S37: Colocalization analysis results.

**Supporting Information 5** MIMIC1—collaborative institutional training initiative.

**Supporting Information 6** MIMIC2—certification.

**Supporting Information 7** Additional supporting information.

**Supporting Information 8** STROBE‐MR checklist fillable.

## Data Availability

This study utilized data from publicly available GWAS summary statistics. Clinical features data were obtained from multiple consortia and biobanks, including the Transdisciplinary Research in Cancer of the Lung (TRICL) consortium, the FinnGen consortium (Release 12), and the UK Biobank GWAS consortium. GWAS summary statistics for LC phenotypes (ieu‐a‐987, ieu‐a‐984, ieu‐a‐989, ieu‐a‐988, C3_BRONCHUS_LUNG_EXALLC, C3_NSCLC_ADENO_EXALLC, C3_NSCLC_SQUAM_EXALLC, and C3_SCLC_EXALLC), COPD (J10_COPD), sepsis (AB1_OTHER_SEPSIS), stroke (C_STROKE), liver cirrhosis (CHIRHEP_NAS), heart failure (I9_HEARTFAIL), hypertension (I9_HYPTENS), ARF (N14_ACUTERENFAIL), CKD (N14_CHRONKIDNEYDIS), Type 1 diabetes, and Type 2 diabetes were accessed through their respective consortia′s public data portals. Plasma pQTL data were obtained from the deCODE genetics study, the UK Biobank Pharma Proteomics Project, and the Fenland study. Outcome data for pneumonia and pneumonia‐related 28‐day mortality were accessed from the UK Biobank GWAS consortium under Study Accessions GCST90281167 and GCST90281172. All additional data supporting this study′s findings are provided within the article and its supporting information file. These data may also be obtained, upon reasonable request, from the corresponding authors. All data were used strictly in accordance with their institutional data use agreements.
