# Supplementary figures and images for: Disentangling Links Between Lung Cancer and Infectious Pneumonia via Real‐World Data and Integrative Genomics
Source: Hum Mutat. 2026 Jan 31;2026:4536781. doi: 10.1155/humu/4536781 (PMC12859732; doi:10.1155/humu/4536781)

# Post-GWAS analysis process

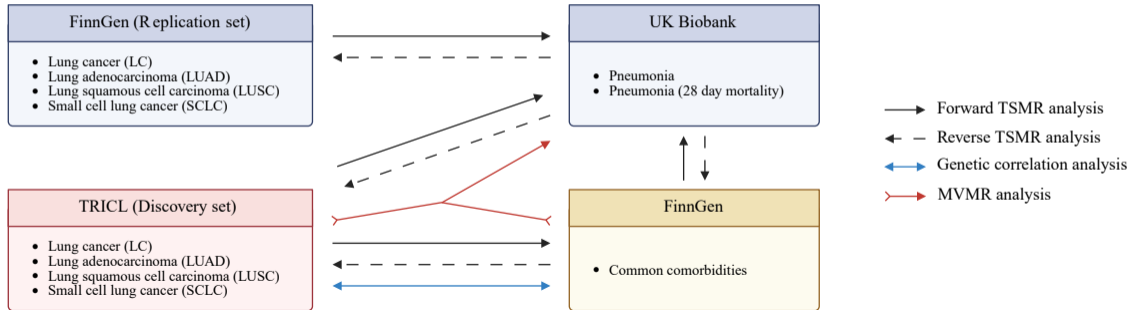

Supplement: Supplementary file 1 — Supporting Information 1 Figure S1: Mendelian randomization principles and mediation analysis process. [file HUMU-2026-4536781-s001.pdf]
