# Supplementary material for: Disentangling Links Between Lung Cancer and Infectious Pneumonia via Real‐World Data and Integrative Genomics
Source: Hum Mutat. 2026 Jan 31;2026:4536781. doi: 10.1155/humu/4536781 (PMC12859732; doi:10.1155/humu/4536781)
Supplement: Supplementary file 2 — Supporting Information 2 Figure S2: Post‐GWAS analysis process. [file HUMU-2026-4536781-s003.pdf]

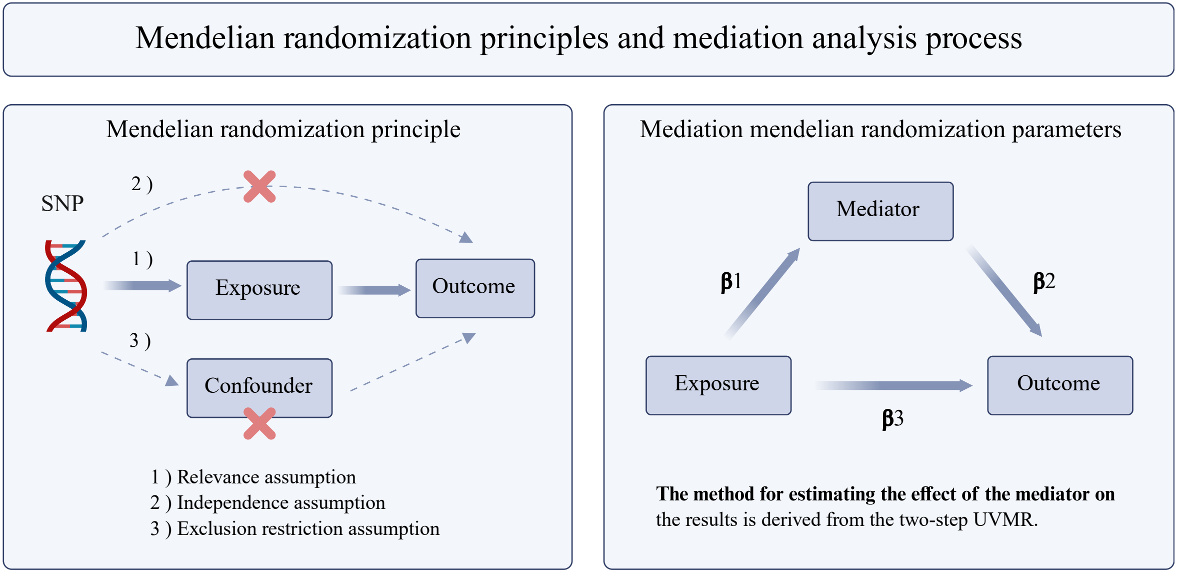
Mendelian randomization principles and mediation analysis process
Mendelian randomization principle
SNP
2)
1)
3)
Exposure
Outcome
Confounder
1) Relevance assumption
2) Independence assumption
3) Exclusion restriction assumption
Mediation mendelian randomization parameters
Mediator
β1
β2
Exposure
Outcome
β3
The method for estimating the effect of the mediator on the results is derived from the two-step UVMR.
