# Supplementary material for: Disentangling Links Between Lung Cancer and Infectious Pneumonia via Real‐World Data and Integrative Genomics
Source: Hum Mutat. 2026 Jan 31;2026:4536781. doi: 10.1155/humu/4536781 (PMC12859732; doi:10.1155/humu/4536781)
Supplement: Supplementary file 3 — Supporting Information 3 Figure S3: Violin diagram of expression pattern and differential analysis of potential comorbidity targets in peripheral blood immune cells. [file HUMU-2026-4536781-s002.pdf]

Gene Expression Distribution Across Cell Types with Significance Markers [Log2(TPM+1)]

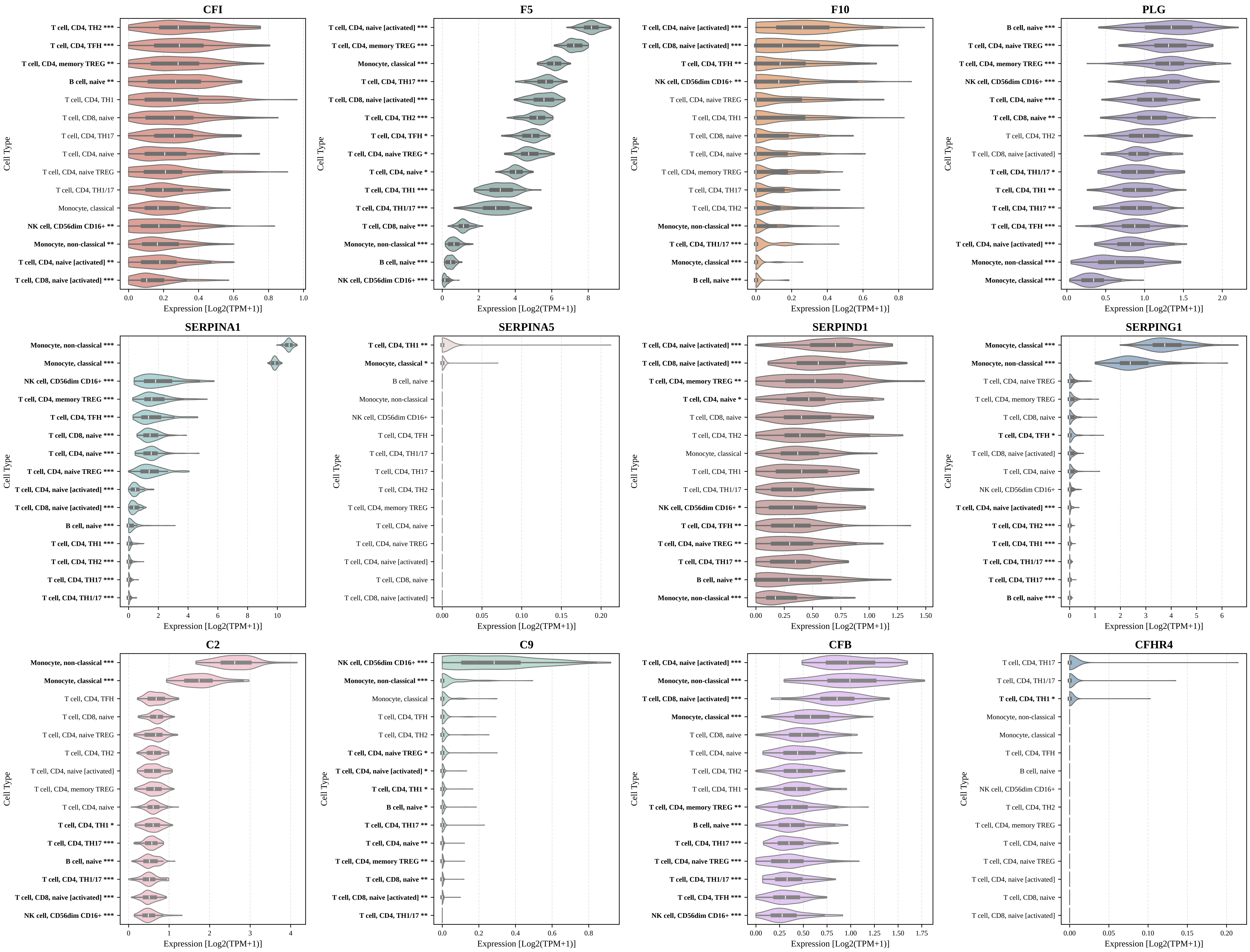

** p<0.05, ** p<0.01, *** p<0.001 (Mann-Whitney U test: each cell type vs. all others)*
