# Supplementary material for: Disentangling Links Between Lung Cancer and Infectious Pneumonia via Real‐World Data and Integrative Genomics
Source: Hum Mutat. 2026 Jan 31;2026:4536781. doi: 10.1155/humu/4536781 (PMC12859732; doi:10.1155/humu/4536781)
Supplement: Supplementary file 5 — Supporting Information 5 MIMIC1—collaborative institutional training initiative. [file HUMU-2026-4536781-s004.pdf]

COLLABORATIVE INSTITUTIONAL TRAINING INITIATIVE (CITI PROGRAM)

COMPLETION REPORT - PART 1 OF 2
COURSEWORK REQUIREMENTS*

* Scores on this Requirements Report (Part 1) reflect quiz completions at the time all requirements for the course were met. The Transcript Report (Part 2) lists more recent quiz scores, including those on optional (supplemental) course elements.

- **Name:** Zhe Chen (ID: 14318014)
- **Institution Affiliation:** Massachusetts Institute of Technology Affiliates (ID: 1912)
- **Institution Email:** czszdx@gmail.com
- **Institution Unit:** Clinical research

- **Curriculum Group:** Human Research
- **Course Learner Group:** Data or Specimens Only Research
- **Stage:** Stage 2 - Refresher Course

- **Record ID:** 68706386
- **Completion Date:** 26-Mar-2025
- **Expiration Date:** 26-Mar-2028
- **Minimum Passing:** 90
- **Reported Score*:** 100

REQUIRED AND ELECTIVE MODULES ONLY	DATE COMPLETED	SCORE
SBE Refresher 1 – Defining Research with Human Subjects (ID: 15029)	26-Mar-2025	2/2 (100%)
SBE Refresher 1 – Privacy and Confidentiality (ID: 15035)	26-Mar-2025	4/4 (100%)
SBE Refresher 1 – Assessing Risk (ID: 15034)	26-Mar-2025	2/2 (100%)
SBE Refresher 1 – Research with Children (ID: 15036)	26-Mar-2025	2/2 (100%)
SBE Refresher 1 – International Research (ID: 15028)	26-Mar-2025	2/2 (100%)
Instructions (ID: 764)	26-Mar-2025	No Quiz
Biomed Refresher 2 – History and Ethical Principles (ID: 511)	26-Mar-2025	3/3 (100%)
Biomed Refresher 2 – Regulations and Process (ID: 512)	26-Mar-2025	2/2 (100%)
Biomed Refresher 2 – SBR Methodologies in Biomedical Research (ID: 515)	26-Mar-2025	4/4 (100%)
Biomed Refresher 2 – Genetics Research (ID: 518)	26-Mar-2025	2/2 (100%)
Biomed Refresher 2 – Records-Based Research (ID: 516)	26-Mar-2025	3/3 (100%)
Biomed Refresher 2 - Populations in Research Requiring Additional Considerations and/or Protections (ID: 519)	26-Mar-2025	1/1 (100%)
Biomed Refresher 2 – HIPAA and Human Subjects Research (ID: 526)	26-Mar-2025	5/5 (100%)
Biomed Refresher 2 – Conflicts of Interest in Research Involving Human Subjects (ID: 17545)	26-Mar-2025	5/5 (100%)

For this Report to be valid, the learner identified above must have had a valid affiliation with the CITI Program subscribing institution identified above or have been a paid Independent Learner.

This document was generated on 26-Mar-2025. Verify at:
www.citiprogram.org/verify/?k8e6a3d74-da37-4f68-9344-12e174381138-68706386

Collaborative Institutional Training Initiative (CITI Program)
101 NE 3rd Avenue
Suite 320
Fort Lauderdale, FL 33301 US

Email: support@citiprogram.org
Phone: 888-529-5929
Web: https://www.citiprogram.org

COLLABORATIVE INSTITUTIONAL TRAINING INITIATIVE (CITI PROGRAM)
COMPLETION REPORT - PART 2 OF 2
COURSEWORK TRANSCRIPT**

** Scores on this Transcript Report (Part 2) reflect the most current quiz completions, including quizzes on optional (supplemental) elements of the course. The Requirements Report (Part 1) lists the reported scores at the time all requirements for the course were met.

- **Name:** Zhe Chen (ID: 14318014)
- **Institution Affiliation:** Massachusetts Institute of Technology Affiliates (ID: 1912)
- **Institution Email:** czszdx@gmail.com
- **Institution Unit:** Clinical research

- **Curriculum Group:** Human Research
- **Course Learner Group:** Data or Specimens Only Research
- **Stage:** Stage 2 - Refresher Course

- **Record ID:** 68706386
- **Current Score**:** 100

REQUIRED, ELECTIVE, AND SUPPLEMENTAL MODULES	MOST RECENT	SCORE
Instructions (ID: 764)	26-Mar-2025	No Quiz
Biomed Refresher 2 – History and Ethical Principles (ID: 511)	26-Mar-2025	3/3 (100%)
Biomed Refresher 2 – Regulations and Process (ID: 512)	26-Mar-2025	2/2 (100%)
Biomed Refresher 2 – SBR Methodologies in Biomedical Research (ID: 515)	26-Mar-2025	4/4 (100%)
SBE Refresher 1 – Defining Research with Human Subjects (ID: 15029)	26-Mar-2025	2/2 (100%)
Biomed Refresher 2 – Records-Based Research (ID: 516)	26-Mar-2025	3/3 (100%)
Biomed Refresher 2 – Genetics Research (ID: 518)	26-Mar-2025	2/2 (100%)
SBE Refresher 1 – Assessing Risk (ID: 15034)	26-Mar-2025	2/2 (100%)
SBE Refresher 1 – Privacy and Confidentiality (ID: 15035)	26-Mar-2025	4/4 (100%)
Biomed Refresher 2 - Populations in Research Requiring Additional Considerations and/or Protections (ID: 519)	26-Mar-2025	1/1 (100%)
SBE Refresher 1 – Research with Children (ID: 15036)	26-Mar-2025	2/2 (100%)
SBE Refresher 1 – International Research (ID: 15028)	26-Mar-2025	2/2 (100%)
Biomed Refresher 2 – HIPAA and Human Subjects Research (ID: 526)	26-Mar-2025	5/5 (100%)
Biomed Refresher 2 – Conflicts of Interest in Research Involving Human Subjects (ID: 17545)	26-Mar-2025	5/5 (100%)

For this Report to be valid, the learner identified above must have had a valid affiliation with the CITI Program subscribing institution identified above or have been a paid Independent Learner.

This document was generated on 26-Mar-2025. Verify at:
www.citiprogram.org/verify/?k8e6a3d74-da37-4f68-9344-12e174381138-68706386

Collaborative Institutional Training Initiative (CITI Program)
101 NE 3rd Avenue
Suite 320
Fort Lauderdale, FL 33301 US

Email: support@citiprogram.org
Phone: 888-529-5929
Web: https://www.citiprogram.org

Collaborative Institutional Training Initiative
