# Supplementary material for: Disentangling Links Between Lung Cancer and Infectious Pneumonia via Real‐World Data and Integrative Genomics
Source: Hum Mutat. 2026 Jan 31;2026:4536781. doi: 10.1155/humu/4536781 (PMC12859732; doi:10.1155/humu/4536781)
Supplement: Supplementary file 6 — Supporting Information 6 MIMIC2—certification. [file HUMU-2026-4536781-s005.pdf]

| | |
|---|---|
| Completion Date | 26-Mar-2025 |
| Expiration Date | 26-Mar-2028 |
| Record ID | 68706386 |

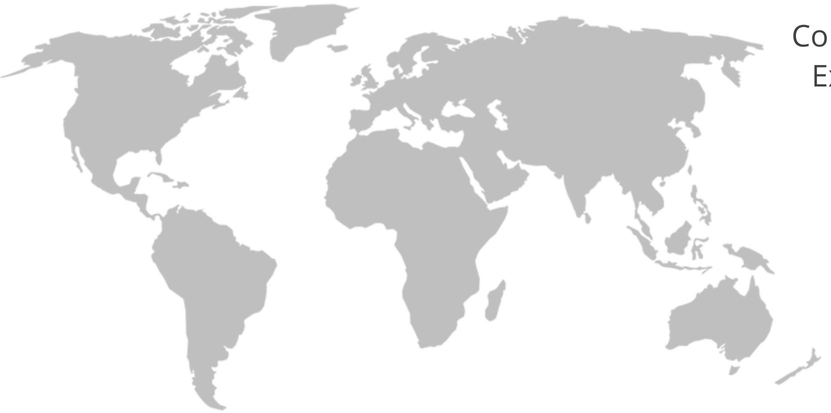

This is to certify that:

**Zhe Chen**

Not valid for renewal of certification through CME.

Has completed the following CITI Program course:

**Human Research**
(Curriculum Group)
**Data or Specimens Only Research**
(Course Learner Group)
**2 - Refresher Course**
(Stage)

Under requirements set by:

**Massachusetts Institute of Technology Affiliates**

Collaborative Institutional Training Initiative

101 NE 3rd Avenue, Suite 320
Fort Lauderdale, FL 33301 US
www.citiprogram.org

Generated on 26-Mar-2025. Verify at www.citiprogram.org/verify/?wca61ffb5-6858-4a04-b722-3a4796f38820-68706386
