# Supplementary material for: Disentangling Links Between Lung Cancer and Infectious Pneumonia via Real‐World Data and Integrative Genomics
Source: Hum Mutat. 2026 Jan 31;2026:4536781. doi: 10.1155/humu/4536781 (PMC12859732; doi:10.1155/humu/4536781)
Supplement: Supplementary file 8 — Supporting Information 8 STROBE‐MR checklist fillable. [file HUMU-2026-4536781-s007.docx]

**STROBE-MR checklist of recommended items to address in reports of Mendelian randomization studies**^1^ ^2^

| **Item No.** | **Section** | **Checklist item** | **Page No.** | **Relevant text from manuscript** |
| --- | --- | --- | --- | --- |
| 1 | **TITLE and ABSTRACT** | Indicate Mendelian randomization (MR) as the study’s design in the title and/or the abstract if that is a main purpose of the study | 2 | Data from two intensive care unit databases were analyzed to assess the association between LC and pneumonia incidence and prognosis from a real-world perspective, with Mendelian randomization (MR) applied to validate causality. |
|  | **INTRODUCTION** |  |  |  |
| 2 | **Background** | Explain the scientific background and rationale for the reported study. What is the exposure? Is a potential causal relationship between exposure and outcome plausible? Justify why MR is a helpful method to address the study question | 4-5 | Although these mechanisms are recognized, most current studies focus on treatment-related pneumonia, and evidence regarding infections directly attributable to LC is limited. To overcome the inherent limitations of observational research, we applied Mendelian randomization (MR) to evaluate the causal effect of LC on pneumonia risk and 28-day mortality. |
| 3 | **Objectives** | State specific objectives clearly, including pre-specified causal hypotheses (if any). State that MR is a method that, under specific assumptions, intends to estimate causal effects | 5 | By leveraging germline genetic variants as instrumental variables, MR offers a robust approach to reduce confounding and improve the validity of causal inference. |
|  | **METHODS** |  |  |  |
| 4 | **Study design and data sources** | Present key elements of the study design early in the article. Consider including a table listing sources of data for all phases of the study. For each data source contributing to the analysis, describe the following: |  |  |
|  | a) | Setting: Describe the study design and the underlying population, if possible. Describe the setting, locations, and relevant dates, including periods of recruitment, exposure, follow-up, and data collection, when available. | NA | NA |
|  | b) | Participants: Give the eligibility criteria, and the sources and methods of selection of participants. Report the sample size, and whether any power or sample size calculations were carried out prior to the main analysis | 7 | The GWAS datasets used in this study were obtained from three large research consortia: TRICL, FinnGen, and UK Biobank, with sample sizes ranging from 23,371 to 500,348 cases. TRICL and FinnGen also provided GWAS data for three histological subtypes of LC. GWAS data for comorbidities were derived from the FinnGen study, and data on infectious pneumonia and 28-day pneumonia-related mortality were obtained from the UK Biobank. For drug target analysis, we incorporated three large-scale pQTL datasets from deCODE, UKBPPP and Fenland. Table S2 provides details of these datasets. |
|  | c) | Describe measurement, quality control and selection of genetic variants | 8 | MR analyses were conducted in accordance with the STROBE-MR guidelines. IVs were selected based on the following criteria: common variants with minor allele frequency > 0.01, genome-wide significance (P < 5 × 10⁻⁸), low linkage disequilibrium (r² < 0.001 within a 1000 kb window), and F-statistics > 10. For pneumonia-related traits and FinnGen small cell lung cancer (SCLC), the significance threshold was relaxed to P < 5 × 10⁻⁷ due to limited power. SNPs showing strong associations with the outcome (P < 5 × 10⁻⁷) were excluded to ensure that valid causal inferences under independence were not violated. |
|  | d) | For each exposure, outcome, and other relevant variables, describe methods of assessment and diagnostic criteria for diseases | NA | GWAS summary project website provides diagnostic criteria. |
|  | e) | Provide details of ethics committee approval and participant informed consent, if relevant | 5 | As the data were fully de-identified, neither informed consent nor institutional ethics approval was required. |
| 5 | **Assumptions** | Explicitly state the three core IV assumptions for the main analysis (relevance, independence and exclusion restriction) as well assumptions for any additional or sensitivity analysis | 8-9 | MR analyses were conducted in accordance with the STROBE-MR guidelines. IVs were selected based on the following criteria: common variants with minor allele frequency > 0.01, genome-wide significance (*P* < 5 × 10⁻⁸), low linkage disequilibrium (r² < 0.001 within a 1000 kb window), and F-statistics > 10. For pneumonia-related traits and FinnGen small cell lung cancer (SCLC), the significance threshold was relaxed to *P* < 5 × 10⁻⁷ due to limited power. SNPs showing strong associations with the outcome (*P* < 5 × 10⁻⁷) were excluded to ensure that valid causal inferences under independence were not violated.  Univariate MR (UVMR) heterogeneity was assessed using Cochran's Q test when two or more IVs were available, while multivariable validity was evaluated using Egger's intercept with three or more IVs. Directionality tests and reverse UVMR analyses were performed to confirm the correct causal direction. The Wald ratio method was applied when only one IV was available, and the inverse variance weighted (IVW) method with a random-effects model was used for multiple instruments to account for heterogeneity. To further reduce potential bias from horizontal pleiotropy, the constrained maximum likelihood and model averaging (cML-MA) method was employed. This approach incorporates Bayesian Information Criterion (BIC) and data perturbation (DP) techniques, with model robustness evaluated through two goodness-of-fit tests. The cML-MA-BIC-DP framework was applied when either test yielded *P* < 0.05. Causal estimates from MR analyses were expressed as ORs, representing the change in outcome risk per unit increase in exposure. For pQTL-based exposures, ORs reflected the effect per standard deviation increase in genetically predicted protein levels. |
| 6 | **Statistical methods: main analysis** | Describe statistical methods and statistics used |  |  |
|  | a) | Describe how quantitative variables were handled in the analyses (i.e., scale, units, model) | 8-9 | Causal estimates from MR analyses were expressed as ORs, representing the change in outcome risk per unit increase in exposure. For pQTL-based exposures, ORs reflected the effect per standard deviation increase in genetically predicted protein levels. |
|  | b) | Describe how genetic variants were handled in the analyses and, if applicable, how their weights were selected | 8 | MR analyses were conducted in accordance with the STROBE-MR guidelines. IVs were selected based on the following criteria: common variants with minor allele frequency > 0.01, genome-wide significance (P < 5 × 10⁻⁸), low linkage disequilibrium (r² < 0.001 within a 1000 kb window), and F-statistics > 10. For pneumonia-related traits and FinnGen small cell lung cancer (SCLC), the significance threshold was relaxed to P < 5 × 10⁻⁷ due to limited power. SNPs showing strong associations with the outcome (P < 5 × 10⁻⁷) were excluded to ensure that valid causal inferences under independence were not violated. |
|  | c) | Describe the MR estimator (e.g. two-stage least squares, Wald ratio) and related statistics. Detail the included covariates and, in case of two-sample MR, whether the same covariate set was used for adjustment in the two samples | 8-9 | Univariate MR (UVMR) heterogeneity was assessed using Cochran's Q test when two or more IVs were available, while multivariable validity was evaluated using Egger's intercept with three or more IVs. Directionality tests and reverse UVMR analyses were performed to confirm the correct causal direction. The Wald ratio method was applied when only one IV was available, and the inverse variance weighted (IVW) method with a random-effects model was used for multiple instruments to account for heterogeneity. To further reduce potential bias from horizontal pleiotropy, the constrained maximum likelihood and model averaging (cML-MA) method was employed. This approach incorporates Bayesian Information Criterion (BIC) and data perturbation (DP) techniques, with model robustness evaluated through two goodness-of-fit tests. The cML-MA-BIC-DP framework was applied when either test yielded *P* < 0.05. Causal estimates from MR analyses were expressed as ORs, representing the change in outcome risk per unit increase in exposure. For pQTL-based exposures, ORs reflected the effect per standard deviation increase in genetically predicted protein levels. |
|  | d) | Explain how missing data were addressed | NA | NA |
|  | e) | If applicable, indicate how multiple testing was addressed |  | MR analyses were considered statistically significant at *P* < 0.05. In UVMR, associations passing Bonferroni correction (0.05 divided by the number of exposures multiplied by outcomes) were regarded as having strong causal evidence. MR estimates from different LC datasets were combined by meta-analysis. Fixed-effects results were reported as the main findings, with heterogeneity assessed across datasets. When I² > 50% and *P* < 0.05, and the fixed- and random-effects models yielded different results, estimates from the random-effects model were also reported to ensure robustness. |
| 7 | **Assessment of assumptions** | Describe any methods or prior knowledge used to assess the assumptions or justify their validity | 8-9 | Univariate MR (UVMR) heterogeneity was assessed using Cochran's Q test when two or more IVs were available, while multivariable validity was evaluated using Egger's intercept with three or more IVs. Directionality tests and reverse UVMR analyses were performed to confirm the correct causal direction. The Wald ratio method was applied when only one IV was available, and the inverse variance weighted (IVW) method with a random-effects model was used for multiple instruments to account for heterogeneity. To further reduce potential bias from horizontal pleiotropy, the constrained maximum likelihood and model averaging (cML-MA) method was employed. This approach incorporates Bayesian Information Criterion (BIC) and data perturbation (DP) techniques, with model robustness evaluated through two goodness-of-fit tests. The cML-MA-BIC-DP framework was applied when either test yielded *P* < 0.05. Causal estimates from MR analyses were expressed as ORs, representing the change in outcome risk per unit increase in exposure. For pQTL-based exposures, ORs reflected the effect per standard deviation increase in genetically predicted protein levels. **Figusure S2**. |
| 8 | **Sensitivity analyses and additional analyses** | Describe any sensitivity analyses or additional analyses performed (e.g. comparison of effect estimates from different approaches, independent replication, bias analytic techniques, validation of instruments, simulations) | 8-9 | Univariate MR (UVMR) heterogeneity was assessed using Cochran's Q test when two or more IVs were available, while multivariable validity was evaluated using Egger's intercept with three or more IVs. Directionality tests and reverse UVMR analyses were performed to confirm the correct causal direction. The Wald ratio method was applied when only one IV was available, and the inverse variance weighted (IVW) method with a random-effects model was used for multiple instruments to account for heterogeneity. To further reduce potential bias from horizontal pleiotropy, the constrained maximum likelihood and model averaging (cML-MA) method was employed. This approach incorporates Bayesian Information Criterion (BIC) and data perturbation (DP) techniques, with model robustness evaluated through two goodness-of-fit tests. The cML-MA-BIC-DP framework was applied when either test yielded *P* < 0.05. Causal estimates from MR analyses were expressed as ORs, representing the change in outcome risk per unit increase in exposure. For pQTL-based exposures, ORs reflected the effect per standard deviation increase in genetically predicted protein levels. |
| 9 | **Software and pre-registration** |  |  |  |
|  | a) | Name statistical software and package(s), including version and settings used | 7, 9 | In addition, we performed subgroup analyses to explore effect heterogeneity and potential interactions. All statistical analyses were completed using R software (version 4.3.3). MVMR was conducted to estimate the independent effects of multiple exposures on a single outcome, using the “MVMR” package with exposure covariance matrices derived from LDSC. To account for pleiotropy, the cML-MA method implemented in the “MVMRcML” package was applied. When Egger’s intercept indicated significant pleiotropy, results from the cML-MA-BIC-DP approach were reported. |
|  | b) | State whether the study protocol and details were pre-registered (as well as when and where) | 8-9 | Univariate MR (UVMR) heterogeneity was assessed using Cochran's Q test when two or more IVs were available, while multivariable validity was evaluated using Egger's intercept with three or more IVs. Directionality tests and reverse UVMR analyses were performed to confirm the correct causal direction. The Wald ratio method was applied when only one IV was available, and the inverse variance weighted (IVW) method with a random-effects model was used for multiple instruments to account for heterogeneity. To further reduce potential bias from horizontal pleiotropy, the constrained maximum likelihood and model averaging (cML-MA) method was employed. This approach incorporates Bayesian Information Criterion (BIC) and data perturbation (DP) techniques, with model robustness evaluated through two goodness-of-fit tests. The cML-MA-BIC-DP framework was applied when either test yielded *P* < 0.05. Causal estimates from MR analyses were expressed as ORs, representing the change in outcome risk per unit increase in exposure. For pQTL-based exposures, ORs reflected the effect per standard deviation increase in genetically predicted protein levels.  MVMR was conducted to estimate the independent effects of multiple exposures on a single outcome, using the “MVMR” package with exposure covariance matrices derived from LDSC. To account for pleiotropy, the cML-MA method implemented in the “MVMRcML” package was applied. When Egger’s intercept indicated significant pleiotropy, results from the cML-MA-BIC-DP approach were reported. |
|  | **RESULTS** |  |  |  |
| 10 | **Descriptive data** |  |  |  |
|  | a) | Report the numbers of individuals at each stage of included studies and reasons for exclusion. Consider use of a flow diagram | 5-6 | This study integrated cross-sectional, cohort, and post-GWAS analyses to evaluate the impact of LC on the onset and short-term outcomes of infectious pneumonia, and to investigate the underlying genetic mechanisms. **Figure 1** outlines the study design, and **Figure S1** details the post-GWAS analytical workflow. All datasets used in this study were publicly available and accessed in compliance with their data use agreements. As the data were fully de-identified, neither informed consent nor institutional ethics approval was required. The design and analytical procedures of these datasets were rigorously developed in accordance with the STROBE (Strengthening the Reporting of Observational Studies in Epidemiology) reporting framework. |
|  | b) | Report summary statistics for phenotypic exposure(s), outcome(s), and other relevant variables (e.g. means, SDs, proportions) | 7 | The GWAS datasets used in this study were obtained from three large research consortia: TRICL, FinnGen, and UK Biobank, with sample sizes ranging from 23,371 to 500,348 cases. TRICL and FinnGen also provided GWAS data for three histological subtypes of LC. GWAS data for comorbidities were derived from the FinnGen study, and data on infectious pneumonia and 28-day pneumonia-related mortality were obtained from the UK Biobank. For drug target analysis, we incorporated three large-scale pQTL datasets from deCODE, UKBPPP and Fenland. Table S2 provides details of these datasets. |
|  | c) | If the data sources include meta-analyses of previous studies, provide the assessments of heterogeneity across these studies | NA | NA |
|  | d) | For two-sample MR:  i.  Provide justification of the similarity of the genetic variant-exposure associations between the exposure and outcome samples  ii.  Provide information on the number of individuals who overlap between the exposure and outcome studies | 13 | To investigate whether shared biological mechanisms between LC and common comorbidities might confound its effect on pneumonia, we conducted genetic correlation analyses using LDSC and HDL methods. To minimize sample overlap and ensure population independence, we integrated comorbidity data from FinnGen with LC data from TRICL and applied LDSC analysis with intercept correction. After Bonferroni correction (*P* < 0.05/40), significant genetic correlations were observed between COPD and LC-related traits, as well as between LUSC and sepsis (**Figure 3A**). Other comorbidities showed suggestive levels of genetic correlation with LC (**Table S22**). |
| 11 | **Main results** |  |  |  |
|  | a) | Report the associations between genetic variant and exposure, and between genetic variant and outcome, preferably on an interpretable scale | 11-12 | To overcome the inherent limitations of observational studies in identifying causal associations, we used a genetic agent-based UVMR approach to validate the above association findings. Detailed characteristics of the IVs are shown in Table S19. |
|  | b) | Report MR estimates of the relationship between exposure and outcome, and the measures of uncertainty from the MR analysis, on an interpretable scale, such as odds ratio or relative risk per SD difference | 12 | In the TRICL dataset, genetically predicted overall LC (OR = 1.122, 95% CI: 1.036-1.215, P = 0.005) and SCLC (OR = 1.122, 95% CI: 1.028-1.225, P = 0.010) were associated with an increased risk of pneumonia, while no significant associations were observed for non-small cell lung cancer subtypes. In contrast, FinnGen data showed that lung adenocarcinoma (LUAD) (OR = 1.161, 95% CI: 1.073-1.256, P < 0.001) and lung squamous cell carcinoma (LUSC) (OR = 1.153, 95% CI: 1.077-1.235, P < 0.001) were significantly associated with increased pneumonia risk, while overall LC and SCLC did not show significant associations. Regarding short-term prognosis, both datasets demonstrated a significant association between overall LC (TRICL: OR = 1.204, 95% CI: 1.060-1.367, P = 0.004; FinnGen: OR = 1.248, 95% CI: 1.049-1.484, P = 0.013) and LUSC (TRICL: OR = 1.129, 95% CI: 1.012-1.260, P = 0.030; FinnGen: OR = 1.214, 95% CI: 1.016-1.450, P = 0.033) were associated with increased 28-day mortality in patients with pneumonia. Additionally, FinnGen data showed a significant association between LUAD and short-term mortality (OR = 1.291, 95% CI: 1.052-1.583, P = 0.014), but this finding was not validated in the TRICL dataset. Unlike the other subtypes, SCLC did not show a significant association with mortality in either data. |
|  | c) | If relevant, consider translating estimates of relative risk into absolute risk for a meaningful time period | NA | NA |
|  | d) | Consider plots to visualize results (e.g. forest plot, scatterplot of associations between genetic variants and outcome versus between genetic variants and exposure) | NA | Figure 3. |
| 12 | **Assessment of assumptions** |  |  |  |
|  | a) | Report the assessment of the validity of the assumptions | 12 | Sensitivity analyses using the cML-MA method produced consistent results in both effect direction and significance, aligning with estimates from the Wald ratio and IVW methods. The MR-Egger intercept test did not detect directional pleiotropy, suggesting limited bias from horizontal pleiotropy (Table S20). |
|  | b) | Report any additional statistics (e.g., assessments of heterogeneity across genetic variants, such as *I^2^*, Q statistic or E-value) | NA | Supplementary tables. |
| 13 | **Sensitivity analyses and additional analyses** |  |  |  |
|  | a) | Report any sensitivity analyses to assess the robustness of the main results to violations of the assumptions | 12 | Sensitivity analyses using the cML-MA method produced consistent results in both effect direction and significance, aligning with estimates from the Wald ratio and IVW methods. The MR-Egger intercept test did not detect directional pleiotropy, suggesting limited bias from horizontal pleiotropy (Table S20). |
|  | b) | Report results from other sensitivity analyses or additional analyses | 12-13 | A meta-analysis integrating the results of the two datasets further supported the presence of significant causal associations, indicating that genetic prediction of overall LC (OR = 1.103, 95% CI: 1.031-1.181, *P* = 0.004), LUAD (OR = 1.098, 95% CI: 1.044-1.155, *P* < 0.001) and LUSC (OR = 1.091, 95% CI: 1.041-1.144, *P* < 0.001) were all associated with an increased pneumonia risk. All associations exceeded the Bonferroni-corrected significance threshold (*P* < 0.05/8). However, the association of LUSC with pneumonia did not reach statistical significance under the random-effects model. SCLC showed a suggestive but non-significant association with the risk of pneumonia (OR = 1.096, 95% CI: 1.017-1.181, *P* = 0.016). Further pooled analysis showed that LC (OR = 1.219, 95% CI: 1.100-1.350, *P* < 0.001), LUSC (OR = 1.152, 95% CI: 1.049-1.264, *P* = 0.003) and LUAD (OR = 1.133, 95% CI: 1.028-1.249, *P* = 0.012) were significantly associated with an increased risk of pneumonia-related short-term mortality. However, after Bonferroni correction, only the association between overall LC and LUSC remained statistically significant, whereas the adverse prognostic effect of LUAD was not confirmed under the random-effects model (**Table 1 and Table S21**). |
|  | c) | Report any assessment of direction of causal relationship (e.g., bidirectional MR) | 13 | Directionality tests supported a causal effect of LC on pneumonia outcomes, and reverse UVMR analysis found no evidence of reverse causality. These findings provide genetic support for a causal relationship between LC and both increased pneumonia risk and worse short-term outcomes, while also highlighting subtype-specific differences. |
|  | d) | When relevant, report and compare with estimates from non-MR analyses | NA | NA |
|  | e) | Consider additional plots to visualize results (e.g., leave-one-out analyses) | NA | NA |
|  | **DISCUSSION** |  |  |  |
| 14 | **Key results** | Summarize key results with reference to study objectives | 16 | This study systematically explored the relationship between LC and infectious pneumonia from multiple perspectives. Analyses based on two independent intensive care unit databases showed that LC was significantly associated with an increased risk of pneumonia and poor short-term prognosis. MR analyses further supported these findings and suggested that different LC subtypes may have different causal effects. By integrating comorbidity-associated genomic data, we identified genetic similarities between LC and a number of common diseases, particularly COPD. However, this genetic overlap did not substantially alter the independent causal effect of LC on pneumonia. Notably, we identified several potential causally mediation pathways, among which COPD appeared to be a key feature linking LC to the pneumonia phenotype. Further drug target analysis identified common pathogenic pathways and molecular targets that may be involved in COPD, LC and pneumonia. These findings deepen our understanding of disease interactions and provide new directions for synergistic therapeutic strategies. |
| 15 | **Limitations** | Discuss limitations of the study, taking into account the validity of the IV assumptions, other sources of potential bias, and imprecision. Discuss both direction and magnitude of any potential bias and any efforts to address them | 19-20 | However, there are some limitations of this study. Although MR is a powerful tool for studying causality, it is still susceptible to horizontal pleiotropy and may introduce bias even after rigorous sensitivity testing. Furthermore, although we used PSM and multiple sensitivity analyses to minimize these effects, retrospective designs can still lead to information bias and selection bias. Further, our genetic analyses were primarily based on populations of European ancestry, limiting the generalizability of the findings. Furthermore, given the complex pattern of comorbidities involved in this study, the actual therapeutic effects of the identified core targets in comorbidity management have not been fully established. Future studies should incorporate experimental interventions and explore strategies that consider potential COPD interactions while balancing infection control with ongoing antitumor therapy to guide clinical decision making and improve the prognosis of patients with LC comorbidities. |
| 16 | **Interpretation** |  |  |  |
|  | a) | Meaning: Give a cautious overall interpretation of results in the context of their limitations and in comparison with other studies | 16-17 | The incidence of pneumonia in LC patients can be as high as 53% during antitumor therapy, and the mechanisms involved are often complex. Taking central LC as an example, it can cause airway obstruction and impair the mucus cilia clearance function, which makes patients prone to secondary infections and then develop serious complications such as pyothorax, lung abscess or fistula formation. In addition, tumor-associated immunosuppression and malignancy significantly increase the susceptibility to opportunistic infections. More importantly, a variety of antitumor treatments themselves can directly contribute to the development of pneumonia, including: chemotherapy- or targeted therapy-induced neutropenia, immune checkpoint inhibitor-associated immune-mediated lung injury, treatment-associated interstitial pneumonia, and radiation pneumonia. Although previous studies have reported an increased incidence of pneumonia in patients with LC, high-quality causal evidence remains lacking and there is limited focus on short-term prognosis in this population. In contrast, the present study used multiple analytical approaches to provide reliable evidence supporting the key causal role of LC in the development and progression of pneumonia. These findings highlight an important clinical dilemma. In current clinical practice, antitumor therapy is usually suspended during pneumonia episodes to reduce the risk of pulmonary toxicity. However, such therapeutic interruptions may accelerate tumor progression and ultimately affect patient prognosis. This dual role exemplifies the inherent complexity of managing comorbidities. Our study provides strong evidence for this intricate relationship and provides a theoretical basis for the development of an integrated treatment strategy that balances tumor control with effective pneumonia management. |
|  | b) | Mechanism: Discuss underlying biological mechanisms that could drive a potential causal relationship between the investigated exposure and the outcome, and whether the gene-environment equivalence assumption is reasonable. Use causal language carefully, clarifying that IV estimates may provide causal effects only under certain assumptions | 18-19 | To enhance the translational potential of this study, we found that complement and coagulation cascade pathways were significantly enriched in the short-term adverse outcomes of LC, COPD, and infectious pneumonia, suggesting that these diseases may share the same pathogenic mechanisms. This finding is highly consistent with previous studies. For example, it has been shown that tumor cells can produce complement components such as C2. In this context, macrophages may secrete C1q, which initiates the activation of the classical complement cascade reaction and thus promotes tumor progression. C2 is also involved in the pathogenesis of childhood multisystemic inflammatory syndrome and COVID-19. In the present study, we further found that the involvement of CFB and *SERPINA1* in infectious pneumonia may be driven by shared germline causal variation, suggesting that these complement-related genes may increase susceptibility to lung infections through genetic mechanisms. In support of this, previous studies have shown that M28 family peptidases can activate training immunity against methicillin-resistant S. aureus infection via the CFB-C3a-C3aR-HIF1α signaling axis by inducing choline depletion and phosphatidylcholine accumulation. In a CFB-deficient mouse model of pneumonia, enhanced extrapulmonary bacterial dissemination and increased inflammatory response were observed, leading to decreased survival. CFB has also been reported to inhibit malignant progression of LC through downregulation of the Ras/MAPK signaling pathway, which is highly consistent with our protein-disease directional findings. Meanwhile, *SERPINA1*, encoding the serine protease inhibitor α-1 antitrypsin, has been proposed as a potential biomarker of poor prognosis in COVID-19 and has been closely associated with infection-associated multi-organ dysfunction. Its deficiency is a recognized factor in the development of COPD, further supporting our results. |
|  | c) | Clinical relevance: Discuss whether the results have clinical or public policy relevance, and to what extent they inform effect sizes of possible interventions | 17-18 | Genetic association analyses indicated that COPD may share more genetic loci with LC, which highlights the genetic basis for the combined treatment of COPD and LC. In addition, mediation analyses revealed a mutually reinforcing interaction between LC and COPD in promoting pneumonogenesis and increasing pneumonia-related mortality. Previous MR studies exploring COPD and LC have usually failed to clearly distinguish the direction of causality, partly because of the difficulty in clarifying causality from clinical associations. Although chronic lung inflammation in patients with COPD is known to increase the risk of lung malignancy over time, the limited survival time of LC patients may mask the development of subsequent COPD. Some investigators have suggested that LC may be associated with worsening COPD symptoms, but the causal relationship remains unclear. Taken together, our findings suggest that COPD and LC may engage in a positive feedback loop in promoting the development and progression of infectious pneumonia, providing a new theoretical basis for integrated therapeutic interventions. |
| 17 | **Generalizability** | Discuss the generalizability of the study results (a) to other populations, (b) across other exposure periods/timings, and (c) across other levels of exposure | 19-20 | However, there are some limitations of this study. Although MR is a powerful tool for studying causality, it is still susceptible to horizontal pleiotropy and may introduce bias even after rigorous sensitivity testing. Furthermore, although we used PSM and multiple sensitivity analyses to minimize these effects, retrospective designs can still lead to information bias and selection bias. Further, our genetic analyses were primarily based on populations of European ancestry, limiting the generalizability of the findings. Furthermore, given the complex pattern of comorbidities involved in this study, the actual therapeutic effects of the identified core targets in comorbidity management have not been fully established. Future studies should incorporate experimental interventions and explore strategies that consider potential COPD interactions while balancing infection control with ongoing antitumor therapy to guide clinical decision making and improve the prognosis of patients with LC comorbidities. |
|  | **OTHER INFORMATION** |  |  |  |
| 18 | **Funding** | Describe sources of funding and the role of funders in the present study and, if applicable, sources of funding for the databases and original study or studies on which the present study is based | 21 | This work was supported by the National Natural Science Foundation of China (No. 82002454) and the Medical Scientific Research Project of Jiangsu Health Commission (No. ZD2021011). |
| 19 | **Data and data sharing** | Provide the data used to perform all analyses or report where and how the data can be accessed, and reference these sources in the article. Provide the statistical code needed to reproduce the results in the article, or report whether the code is publicly accessible and if so, where | 21 | All additional data supporting this study’s findings are provided within the article and its supplementary information file. These data may also be obtained, upon reasonable request, from the corresponding author. |
| 20 | **Conflicts of Interest** | All authors should declare all potential conflicts of interest | 21 | The authors declare that they have no competing interests. |

This checklist is copyrighted by the Equator Network under the Creative Commons Attribution 3.0 Unported (CC BY 3.0) license.

1. Skrivankova VW, Richmond RC, Woolf BAR, Yarmolinsky J, Davies NM, Swanson SA, et al. Strengthening the Reporting of Observational Studies in Epidemiology using Mendelian Randomization (STROBE-MR) Statement. JAMA. 2021;under review.

2. Skrivankova VW, Richmond RC, Woolf BAR, Davies NM, Swanson SA, VanderWeele TJ, et al. Strengthening the Reporting of Observational Studies in Epidemiology using Mendelian Randomisation (STROBE-MR): Explanation and Elaboration. BMJ. 2021;375:n2233.
